# Whole genome sequencing for the diagnosis of neurological repeat expansion disorders in the UK: a retrospective diagnostic accuracy and prospective clinical validation study

**DOI:** 10.1016/S1474-4422(21)00462-2

**Published:** 2022-03

**Authors:** Kristina Ibañez, James Polke, R Tanner Hagelstrom, Egor Dolzhenko, Dorota Pasko, Ellen Rachel Amy Thomas, Louise C Daugherty, Dalia Kasperaviciute, Katherine R Smith, WGS for Neurological Diseases Group, Zandra C Deans, Sue Hill, Tom Fowler, Richard H Scott, John Hardy, Patrick F Chinnery, Henry Houlden, Augusto Rendon, Mark J Caulfield, Michael A Eberle, Ryan J Taft, Arianna Tucci, Genomics England Research Consortium, John C. Ambrose, Prabhu Arumugam, Marta Bleda, Freya Boardman-Pretty, Jeanne M. Boissiere, Christopher R. Boustred, Clare E.H. Craig, Anna de Burca, Andrew Devereau, Greg Elgar, Rebecca E. Foulger, Pedro Furió-Tarí, Joanne Hackett, Dina Halai, Angela Hamblin, Shirley Henderson, James Holman, Tim J.P. Hubbard, Rob Jackson, Louise J. Jones, Melis Kayikci, Lea Lahnstein, Kay Lawson, Sarah E.A. Leigh, Ivonne U.S. Leong, Javier F. Lopez, Fiona Maleady-Crowe, Joanne Mason, Michael Mueller, Nirupa Murugaesu, Chris A. Odhams, Daniel Perez-Gil, Dimitris Polychronopoulos, John Pullinger, Tahrima Rahim, Pablo Riesgo-Ferreiro, Tim Rogers, Mina Ryten, Kevin Savage, Kushmita Sawant, Afshan Siddiq, Alexander Sieghart, Damian Smedley, Alona Sosinsky, William Spooner, Helen E. Stevens, Alexander Stuckey, Razvan Sultana, Simon R. Thompson, Carolyn Tregidgo, Emma Walsh, Sarah A. Watters, Matthew J. Welland, Eleanor Williams, Katarzyna Witkowska, Suzanne M. Wood, Magdalena Zarowiecki

**Affiliations:** a Genomics England, Queen Mary University of London, London, UK; b William Harvey Research Institute, Queen Mary University of London, London, UK; c Neurogenetics Unit, National Hospital for Neurology and Neurosurgery, London, UK; d Illumina, San Diego, CA, USA; e Healx, Cambridge, UK; f Genomics Quality Assessment, Department of Laboratory Medicine, Royal Infirmary of Edinburgh, Edinburgh, UK; g NHS England and NHS Improvement, London, UK; h Department of Genetics and Genomic Medicine, UCL Great Ormond Street Institute of Child Health, London, UK; i Department of Neurodegenerative Disorders, Institute of Neurology, University College London, London, UK; j Department of Neuromuscular Diseases, Institute of Neurology, University College London, London, UK; k MRC Mitochondrial Biology Unit and Department of ClinicalNeurosciences, University of Cambridge, Cambridge, UK

## Abstract

**Background:**

Repeat expansion disorders affect about 1 in 3000 individuals and are clinically heterogeneous diseases caused by expansions of short tandem DNA repeats. Genetic testing is often locus-specific, resulting in underdiagnosis of people who have atypical clinical presentations, especially in paediatric patients without a previous positive family history. Whole genome sequencing is increasingly used as a first-line test for other rare genetic disorders, and we aimed to assess its performance in the diagnosis of patients with neurological repeat expansion disorders.

**Methods:**

We retrospectively assessed the diagnostic accuracy of whole genome sequencing to detect the most common repeat expansion loci associated with neurological outcomes (*AR, ATN1, ATXN1, ATXN2, ATXN3, ATXN7, C9orf72, CACNA1A, DMPK, FMR1, FXN, HTT*, and *TBP*) using samples obtained within the National Health Service in England from patients who were suspected of having neurological disorders; previous PCR test results were used as the reference standard. The clinical accuracy of whole genome sequencing to detect repeat expansions was prospectively examined in previously genetically tested and undiagnosed patients recruited in 2013–17 to the 100 000 Genomes Project in the UK, who were suspected of having a genetic neurological disorder (familial or early-onset forms of ataxia, neuropathy, spastic paraplegia, dementia, motor neuron disease, parkinsonian movement disorders, intellectual disability, or neuromuscular disorders). If a repeat expansion call was made using whole genome sequencing, PCR was used to confirm the result.

**Findings:**

The diagnostic accuracy of whole genome sequencing to detect repeat expansions was evaluated against 793 PCR tests previously performed within the NHS from 404 patients. Whole genome sequencing correctly classified 215 of 221 expanded alleles and 1316 of 1321 non-expanded alleles, showing 97·3% sensitivity (95% CI 94·2–99·0) and 99·6% specificity (99·1–99·9) across the 13 disease-associated loci when compared with PCR test results. In samples from 11 631 patients in the 100 000 Genomes Project, whole genome sequencing identified 81 repeat expansions, which were also tested by PCR: 68 were confirmed as repeat expansions in the full pathogenic range, 11 were non-pathogenic intermediate expansions or premutations, and two were non-expanded repeats (16% false discovery rate).

**Interpretation:**

In our study, whole genome sequencing for the detection of repeat expansions showed high sensitivity and specificity, and it led to identification of neurological repeat expansion disorders in previously undiagnosed patients. These findings support implementation of whole genome sequencing in clinical laboratories for diagnosis of patients who have a neurological presentation consistent with a repeat expansion disorder.

**Funding:**

Medical Research Council, Department of Health and Social Care, National Health Service England, National Institute for Health Research, and Illumina.

## Introduction

Despite recent advances in our understanding of the genetic basis of rare neurological disorders, up to 70% of patients with such disorders remain genetically undiagnosed.^1^, ^2^, ^3^ In part, this is due to the technical challenges of testing for complex and repetitive genetic variants, including repeat expansions; such expansions are estimated to affect about 1 in 3000 people (appendix p 1), and are the leading cause of more than 40 neurogenetic disorders,^4^ including Huntington's disease and fragile X syndrome. Repeat expansion disorders are clinically and genetically heterogeneous, and a repeat expansion can be associated with various diseases. For example, expansions in *C9orf72* can present as either amyotrophic lateral sclerosis or frontotemporal dementia.^5^ Repeat expansions in different loci can also yield similar phenotypic features, making them difficult to distinguish clinically: repeat expansions in at least ten spinocerebellar ataxia genes frequently present as adult-onset ataxia,^6^ and those in *C9orf72* and *AR* can both cause motor neuron disease.^7^, ^8^

**Research in context**

**Evidence before this study**

We searched PubMed from database inception to Nov 1, 2020, without language restrictions, for studies published in English using the search terms “repeat expansion diseases” OR “short tandem repeat expansion” AND “whole genome sequencing” OR “next generation sequencing”. Although some studies showed that whole genome sequencing can provide additional and unexpected diagnosis, no studies deployed whole genome sequencing to resolve regions with repeat expansions in a clinically validated pipeline. Repeat expansion disorders are estimated to affect about 1 in 3000 people and primarily affect the nervous system. The defining characteristic of these conditions is the expansion of short (3–6 bp) repetitive DNA sequences beyond a pathogenic threshold. These disorders include well known conditions, such as Huntington's disease, as well as *C9orf72*-associated frontal lobe dementia and amyotrophic lateral sclerosis. Repeat expansion disorders show considerable clinical and genetic heterogeneity, with variability in both clinical presentation and genetic pleiotropy. Whole genome sequencing is rapidly transitioning into clinical practice as a mainstay of genetic diagnosis. The overall diagnostic success rate, however, is generally less than 50%, in part due to the technical limitations of sequencing technology. Repeat expansions have historically been undetectable by whole genome sequencing, contributing to underdiagnosis in patients with suspected genetic neurological disorders and limiting the benefit of genomic testing.

**Added value of this study**

Here we report on the diagnostic accuracy and clinical validation of detection of repeat expansions by whole genome sequencing. Our findings show that whole genome sequencing is both sensitive and specific when compared against previously gold-standard tested positive and negative controls, and that it can lead to a diagnosis in previously undiagnosed patients with suspected neurological disorders in the UK 100 000 Genomes Project cohort.

**Implications of all the available evidence**

Findings from this study support the integration of whole genome sequencing for the detection of repeat expansions in routine clinical practice, and provide a foundation for future studies using whole genome sequencing to assess all repeat expansion disorders. Further work will be needed to reduce the false positives in some loci, such as *FMR1*.

Repeat expansion disorders are caused by an increase in the number of repetitive short tandem DNA sequences, and the pathogenicity thresholds for each disorder are locus-specific. The size of expansion varies from fewer than 30 repeats (eg, in *CACNA1A*) to several thousand repeat units (eg, in *FMR1*, *DMPK*, *C9orf72*, and *FXN*, which can extend up to 5 kb in size). Repeat expansions exhibit molecular instability, which can lead to changes in the repeat size (generally increasing in length) across generations and tissues.^4^ In these conditions, an increase in the number of repeats often leads to an earlier onset and more severe disease in successive generations.^4^ Paediatric onset of repeat expansion disorders can present as multisystem syndromes without specific phenotypic signatures,^9^ and children with these disorders are therefore more likely to be underdiagnosed when a family history of repeat expansion disorder is absent than when it is present.^10^, ^11^, ^12^

Laboratory assessment of repeat expansions is typically restricted to targeted molecular assessment of an individual locus guided by the suspected clinical diagnosis using PCR-based or Southern blot methods,^13^ which can be costly and time-consuming. Additionally, due to the varied and overlapping phenotypic features of these disorders, disease-associated repeat expansion loci can remain untested.^14^

Whole genome sequencing is emerging as a first-line diagnostic tool in patients with rare disease^15^ but, until recently, was thought to have limited capability to assess loci containing repeat expansions.^16^ Advances in bioinformatics, however, have made feasible the detection of disease-causing repeat expansions from next-generation sequencing data.^17^, ^18^, ^19^, ^20^, ^21^, ^22^ Here, we report on the diagnostic assessment of a whole genome sequencing approach to detect repeat expansions using retrospective PCR data, and its clinical validation in patients in the 100 000 Genomes Project who had a suspected neurological disorder, undiagnosed with previous genetic testing.

## Methods

### Study design and participants

This evaluation of whole genome sequencing for detection of repeat expansions included both diagnostic accuracy and clinical accuracy assessments. Diagnostic accuracy was evaluated using data from patients who had previously been tested by PCR for repeat expansions known to cause neurological disease.^4^ Patients were identified from two sources: the 100 000 Genomes Project and the Genomic Laboratory based at Cambridge University Hospitals (Cambridge, UK). For both sets of patients, PCR testing had been performed on patient samples by laboratories in the National Health Service (NHS) as part of routine clinical assessment: for samples in the 100 000 Genomes Project, PCR tests were done before recruitment to the project by the University College London Hospital Neurogenetics Laboratory (London, UK); samples with PCR-confirmed repeat expansions were obtained from patients tested by the Genomic Laboratory based at Cambridge. Patients with PCR-positive and PCR-negative test results for repeat expansion disorders were identified for inclusion in our study through laboratory record systems; all patients had given written informed consent for use of their sample for quality assurance and research and training purposes, as part of clinical service optimisation and validation.

Whole genome sequencing of each sample was done at one of two laboratories: Genomics England (Hinxton, UK) for the 100 000 Genomes Project samples (n=254) and the Illumina Clinical Services Labortatory (ICSL; San Diego, CA, USA) for samples obtained by the Genomic Laboratory based at Cambridge (n=150). Overall, this dataset was used for the diagnostic accuracy part of the study, and consisted of PCR and whole genome sequencing data from 404 patients, covering 13 loci that represent the most common neurological repeat expansion disorders: 11 loci associated with ataxia and late-onset neurodegenerative disorders (*HTT*, *AR, ATN1, ATXN1, ATXN2, ATXN3, ATXN7, CACNA1A*, *TBP, C9orf72,* and *FXN*), one locus associated with intellectual disability (*FMR1*), and one locus associated with myotonic dystrophy (*DMPK*). For each locus, PCR test data were available for at least one expanded allele (appendix p 24).

Clinical accuracy was assessed by examining the concordance of repeat expansions, as detected with whole genome sequencing, with suspected clinical diagnosis after PCR confirmation in patients with suspected genetic neurological disorders (familial or early-onset forms of ataxia, neuropathy, spastic paraplegia, dementia, motor neuron disease, parkinsonian movement disorders, intellectual disability, or neuromuscular disorders) recruited to the 100 000 Genomes Project in 2013–17. The 100 000 Genomes Project is a UK programme to assess the value of whole genome sequencing in patients with unmet diagnostic needs in rare disease and cancer. Following ethical approval for the 100 000 Genomes Project by the East of England Cambridge South Research Ethics Committee (reference 14/EE/1112), including for data analysis and return of diagnostic findings to the patients, these patients were recruited by health-care professionals and researchers from 13 Genomic Medicine Centres in England, and were enrolled in the project if they or their guardian provided written consent for their samples and data to be used in research, including this study. Probands and, if feasible, other family members, were enrolled according to eligibility criteria set for specific rare disease conditions (appendix pp 5–11). Patients were recruited to the 100 000 Genomes Project after standard-of-care genetic testing in the NHS, as indicated in the eligibility criteria. Standardised baseline clinical data were recorded using Human Phenotyping Ontology (HPO)^23^ against disease-specific data models.^24^ The disease status of family members, relative to the proband's clinical indication for testing, was also collected.

To identify causative repeat expansions in patients with genetically undiagnosed disease, we tested patients with suspected genetic disorders consistent with a repeat expansion disease. Patients were selected on the basis of concordance of their disease and HPO terms with repeat expansion-associated disorders. Patients’ whole genome sequencing data were interrogated to search for expansions in particular sets of repeats using four different repeat expansion panels according to their clinical characteristics (appendix p 5). The repeat expansions selected for inclusion on these panels are the most common neurological disease-causing repeat expansion loci. Patients with clinical features potentially compatible with more than one repeat expansion disorder were tested on multiple panels.

If a repeat expansion call was made using whole genome sequencing, confirmatory testing by PCR was performed. For each patient with a confirmed repeat expansion, the local clinician was informed of the potentially diagnostic result, and the contribution of the repeat expansion to the patient's clinical features was assessed. For repeat expansions that fully or partially explained the patient's clinical features, a diagnostic report was issued according to local standard procedures.

### Procedures

For the NHS historical samples used in the diagnostic accuracy part of our study, repeat expansions had previously been tested using PCR amplification and fragment analysis. Southern blotting was performed for large C9orf72 expansions. In the clinical accuracy part of our study, repeat expansions detected by whole genome sequencing in patients from the 100 000 Genomes Project were tested by PCR in samples stored in NHS genetic laboratories. Additional details, including primer sequences, are provided in the appendix (pp 2–3, 25–26).

DNA was prepared for whole genome sequencing using TruSeq DNA PCR-Free library preparation, and 150 bp or 125 bp paired-end sequencing was performed on either HiSeq 2000 or HiSeq X platforms at the high-throughput genomes facility for Genomics England, and at the ICSL. Genomes were sequenced to an average depth of 35× (31× to 37×; appendix p 27).

Short-tandem-repeat genotyping was performed using the ExpansionHunter software package version 3.1.2.^25^, ^26^ In brief, ExpansionHunter realigns sequencing reads across a predefined set of short tandem repeats to estimate the size of both alleles from an individual (appendix p 3). ExpansionHunter output includes an estimation of the number of repeat elements, overall size, and confidence limit for each locus assessed. Guidelines from the Association for Medical Pathology and the College of American Pathologists recommend visual inspection of variant calls during routine assessment of high-throughput sequencing variants.^27^ However, short tandem repeat variants cannot be adequately visualised by common visualisation tools such as Integrative Genomics Viewer.^28^ To examine whole genome sequencing data underlying each genotype call, a graph visualisation tool was used, which enables direct visualisation of haplotypes and the corresponding read pileup of ExpansionHunter genotypes (appendix pp 3, 15). Visual inspection of the pileup graph was performed on all whole genome sequencing short tandem repeat calls to confirm that the ExpansionHunter prediction for alleles was entirely contained in each read (ie, the repeat sequence was smaller than the sequencing read length); to confirm the presence of a monoallelic or biallelic expansion; to detect putative false positive calls; and to detect false negative alleles in biallelic repeat expansions, such as *FXN* (appendix pp 4, 16).

ExpansionHunter estimates repeat size from whole genome sequencing data by analysing sequencing reads that fully or partially contain a short tandem repeat. If a short tandem repeat allele is shorter than the read length, ExpansionHunter predicts the exact size; if a short tandem repeat allele is longer than the read length, ExpansionHunter estimates the repeat size within a CI, depending on locus sequence composition, the depth of sequencing, and the quality of sequencing.

### Statistical analysis

We classified repeats as expanded by whole genome sequencing if the size predicted by ExpansionHunter was above the premutation cutoff, or non-expanded if the predicted size was below the cutoff (appendix p 28).

Sensitivity and CIs for whole genome sequencing repeat expansion detection were calculated as the proportion of alleles with expanded repeats among previously PCR-confirmed alleles with expanded repeats. The specificity was estimated as the proportion of non-expanded alleles among previously tested non-expanded repeats by PCR. A full description of the statistical formulae is provided in the appendix (p 1).

To compare repeat sizes by PCR with repeat size estimates by whole genome sequencing, PCR-quantified alleles were compared with repeat sizes predicted by ExpansionHunter for alleles shorter than the read length across all 13 short tandem repeat loci. Concordance was calculated by the percentage of repeat sizes predicted by ExpansionHunter that were in agreement with PCR-quantified size, taking into account the PCR error of plus or minus one repeat. Statistical analysis was performed using R statistical software version 3.6.3.

### Role of the funding source

The study design, patient enrolment, data collection, and sequencing were led by employees of Genomics England and academic researchers. Employees of Illumina performed the sequencing of 150 patient samples as a planned component of the whole genome sequencing diagnostic accuracy study, and developed ExpansionHunter. Employees of Genomics England, acadamic researchers, and coauthors RTH, ED, and MAE performed the analysis and interpretation of repeat expansions in patients recruited to the 100 000 Genomes Project. The funding sources had no role in data interpretation or writing of the report.

## Results

**Figure 1:**
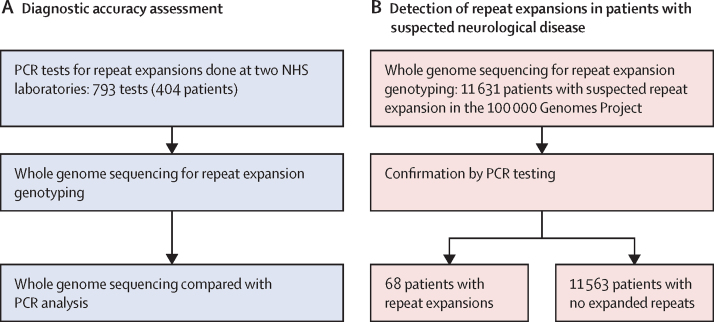
Study flow chart (A) Detection of repeat expansions by whole genome sequencing. (B) Validation in patients who had a suspected neurological disorder, undiagnosed with previous genetic testing. NHS=National Health Service.

**Figure 2:**
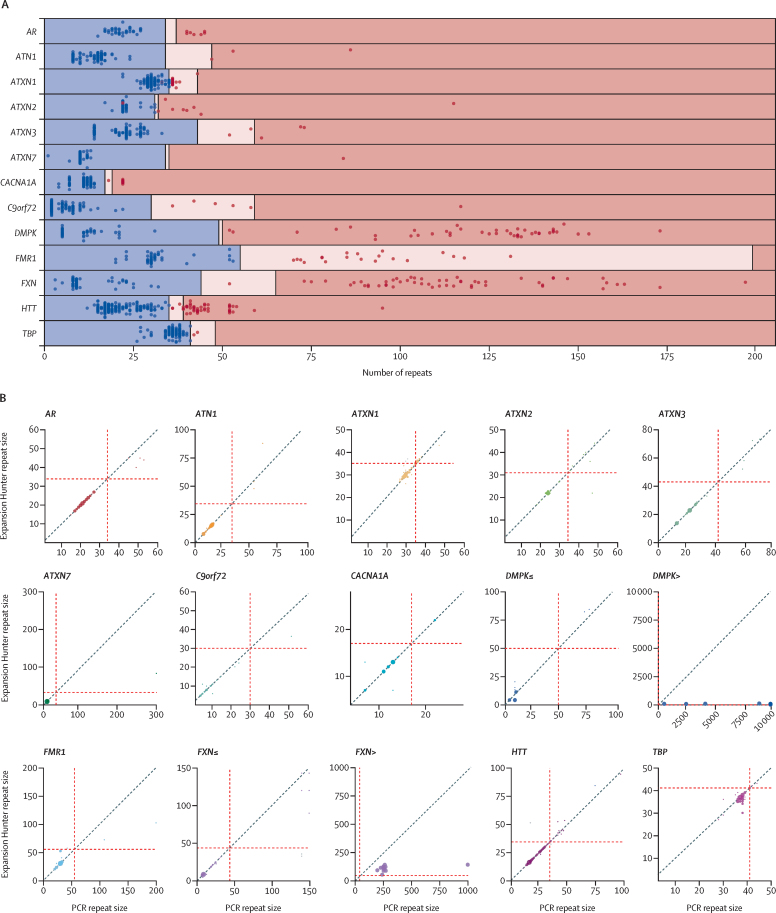
Performance of repeat expansion detection using whole genome sequencing (A) Swim lane plot showing sizes of repeat expansions predicted by ExpansionHunter across 793 expansion calls. Each genome is represented by two points, one corresponding to each allele for each locus, with the exception of those on the X chromosome (ie, *FMR1* and *AR*) in males, for which only one point is shown. Points indicate the repeat length estimated by ExpansionHunter after visual inspection and the colours indicate the repeat size as assessed by PCR (blue represents non-expanded; red represents expanded). The regions are shaded to indicate non-expanded (blue), premutation (pink), and expanded (red) ranges for each gene, as indicated in the appendix (p 28). Blue points in pink or red shaded regions indicate false positives and red points in blue shaded regions indicate false negatives. The individual calls are provided in the appendix (p 27). (B) Repeat size correlation by locus. Bubble plots show PCR repeat sizes on the x axes and ExpansionHunter repeats sizes on y axes, with the size of each dot showing the number of patients with the same repeat size. The grey points visible for *ATXN1*, *FMR1, FXN≤*, and *HTT* represent ExpansionHunter estimations before visual inspection, whereas the corrected ExpansionHunter sizes after visual inspection are in colour. Red dashed lines represent the premutation cutoff for each locus (appendix p 28). *FXN≤* and *DMPK≤* show the repeat size correlation when the the size is less than or equal to the read length (ie, 150 bp). *FXN>* and *DMPK>* show the repeat size correlation when the size is larger than the read length.

The diagnostic accuracy of whole genome sequencing to detect repeat expansions was evaluated against 793 PCR tests previously performed within the NHS from 404 patients (64 patients were tested for more than one repeat; figure 1). Of these tests, 183 were classified as having an expanded repeat and 610 as not having a repeat expansion by PCR, yielding a total of 221 expanded and 1321 non-expanded individual alleles across 13 disease loci (appendix pp 24, 28). Whole genome sequencing correctly classified 215 of 221 expanded alleles and 1316 of 1321 non-expanded alleles compared with PCR test results (appendix pp 27, 29), showing an initial sensitivity of 97·3% (95% CI 94·2–99·0) and specificity of 99·6% (99·1–99·9; table 1). Following the visual correction of all calls based on the quality of the reads, sensitivity increased to 99·1% (96·8–99·9) and specificity to 100% (99·7–100; figure 2A, table 1). Visualisation of the expanded alleles enabled detection of false positive results and reclassification of all false negative alleles in *FXN*, of which only one allele was correctly classified as expanded in samples with biallelic expansions (appendix pp 17, 18).

**Table 1:** Performance of whole genome sequencing in detection of repeat expansions

	**Before visual inspection**	**After visual inspection**
True negative	1316	1321
False positive	5	0
True positive	215	219
False negative	6	2
Specificity, % (95% CI)	99·6% (99·1–99·9)	100% (99·7–100)
Sensitivity, % (95% CI)	97·3% (94·2–99·0)	99·1% (96·8–99·9)
Positive predictive value, % (95% CI)	97·7% (94·7–99·0)	100%
Negative predictive value, % (95% CI)	99·6% (99·0–99·8)	99·9% (99·4–100)
Accuracy, % (95% CI)	99·3% (98·7–99·6)	100% (99·5–100)

Performance based on total number of non-expanded and expanded alleles across all loci tested before and after visual inspection.

Repeat length was quantified by PCR in 509 PCR tests interrogating 945 alleles across 13 repeat expansion loci. Correlations between ExpansionHunter and PCR for repeat sizes shorter and larger than the sequencing read length (ie, 150 bp) are shown in the appendix (appendix p 19). High concordance was observed for repeats shorter than the read length, with 92·7% (836 of 902) agreement between PCR and ExpansionHunter. Locus variability was observed, with high concordance between ExpansionHunter and PCR for *ATXN2*, *ATXN7*, *CACNA1A,* and *HTT*, and low concordance for *DMPK* or *TBP* (appendix p 30). The lengths of alleles larger than the read length were underestimated by ExpansionHunter, which affected the accuracy of calling in *DMPK*, *FMR1*, and *FXN* (figure 2B, appendix pp 19, 31).

Although ExpansionHunter was able to correctly identify large expanded alleles in *FMR1*, *DMPK*, *C9orf72*, and *FXN* (appendix p 29), the predicted size estimates tended to be lower than those obtained by PCR as repeat size increased within the pathogenic range, which affected the ability to distinguish between large and small expansions in *DMPK*, *C9orf72,* and *FXN*, or between full expansions and premutations in *FMR1* (appendix p 31). For example, loci with a PCR-assessed repeat length larger than 200 repeats in *FMR1* and classified as a full mutation had a mean repeat size estimated by ExpansionHunter of 92·6 (SD 17·8; appendix p 31).

To test the ability of repeat expansion detection by whole genome sequencing to resolve the diagnosis of previously tested and genetically undiagnosed patients, we tested 11 631 patients with a suspected genetic neurological disorder recruited to the 100 000 Genomes Project (figure 1). Whole genome sequencing data were evaluated using four different repeat expansion panels according to the patient's clinical features. The numbers of patients tested with each of the four panels are shown in table 2. Overall, we detected and visually confirmed repeat expansions in samples from 105 patients (table 2, appendix pp 20, 33). Of these, 81 samples were available for confirmatory testing by PCR, and 68 were confirmed as having a repeat expansion (0·6% yield): 45 (1·2%) of 3692 in panel A, eight (0·3%) of 2743 in panel B, five (0·6%) of 860 in panel C, and ten (0·1%) of 6731 in panel D. Thirteen of 81 expansion calls were not confirmed as pathogenic repeat expansions (16% false discovery rate). Of these, two were non-expanded alleles in *ATXN1* and *ATXN2*, four were *FMR1* intermediate size calls (appendix p 21), and seven were *FMR1* premutations. Clinical details of the 68 patients with repeat expansions confirmed by PCR, including their clinical presentations, the repeat expansion identified, and the contribution of the repeat expansion to the patient's clinical features are provided in table 3; the HPO terms, repeat size estimated by ExpansionHunter, and whether a diagnostic report has been issued are listed in the appendix (p 33).

**Table 2:** Clinical features and repeat expansion detection in patients from the 100 000 Genomes Project

		**All patients tested**									**Patients with confirmed repeat expansions**		
				Sex									
		Number of patients, n (families, n)	Age, years, median (range)	Male	Female	Mean age at onset, years + months (SD)	Family history, n (%)	Repeat expansion called	Repeat expansion after visual inspection	Repeat expansion tested by PCR	Repeat expansion confirmed, n (families, n)	Mean age at onset, years + months (SD)	Family history, n (%)
Overall		11 631 (10 417)	16 (9–39)	6677 (57%)	4954 (43%)	12+5 (20+3)	3139 (27%)	293	105	81	68 (60)	26+4 (23+9)	29 (48%)
Panel A (*AR, ATN1, ATXN1, ATXN2, ATXN3, ATXN7, C9ORF72, CACNA1A, FMR1, FXN, HTT,* and *TBP)*													
	Hereditary ataxia	1182 (1049)	55 (36–68)	597 (51%)	585 (49%)	35+6 (22+6)	403 (34%)	51	22	19	19 (18)	39+3 (16+0)	9 (50%)
	Hereditary spastic paraplegia	526 (448)	44 (29–60)	275 (52%)	251 (48%)	25+10 (20+0)	221 (42%)	15	8	4	3 (3)	21+0 (0)*	0
	Early-onset and familial Parkinson's disease	520 (508)	57 (50–67)	304 (58%)	216 (42%)	44+0 (13+3)	5 (1%)	16	4	2	2 (2)	36+0 (16+3)	1 (50%)
	Complex parkinsonism	150 (148)	65 (55–72)	85 (57%)	65 (43%)	48+5 (18+10)	31 (21%)	10	3	2	2 (2)	44+6 (0+8)	1 (50%)
	Early-onset dystonia	298 (268)	34 (20–52)	116 (39%)	182 (61%)	22+0 (16+3)	104 (35%)	9	2	0	0	..	0
	Early-onset dementia	151 (145)	63 (58–71)	74 (49%)	77 (51%)	53+11 (13+0)	88 (58%)	17	7	5	4 (4)	48+4 (12+6)	2 (50%)
	Amyotrophic lateral sclerosis	107 (105)	51 (41–67)	69 (64%)	38 (36%)	42+6 (16+4)	19 (18%)	9	8	8	8 (7)	51+2 (15+11)	6 (86%)
	Charcot-Marie-Tooth disease	692 (587)	54 (33–69)	410 (59%)	282 (41%)	31+0 (22+0)	278 (40%)	18	7	4	4 (4)	20+3 (25+9)	1 (25%)
	Ultra-rare undescribed monogenic disorders	62 (55)	44 (28–62)	21 (34%)	41 (66%)	17+9 (20+1)	19 (31%)	5	3	3	3 (2)	31+0 (26+10)	2 (100%)
	Overall panel A	3692 (3305)	55 (41–68)	1954 (53%)	1738 (47%)	34+10 (21+5)	1336 (36%)	150	64	47	45 (42)	38+6 (19+3)	22 (52%)
Panel B (*ATN1, ATXN1, ATXN2, ATXN3, ATXN7, CACNA1A,* and *HTT*)													
	Complex intellectual disability†	2743 (2492)	12 (8–19)	1522 (55%)	1221 (45%)	1+7 (5+3)	528 (19%)	14	9	8	8 (8)	0+6 (1+0)	1 (13%)
Panel C (*DMPK*)													
	Congenital myopathy	471 (422)	21 (13–44)	259 (55%)	212 (45%)	11+1 (18+0)	116 (25%)	1	1	1	1 (1)	30+0 (0)	1 (100%)
	Distal myopathies	185 (167)	58 (42–68)	120 (65%)	65 (35%)	36+11 (22+3)	52 (28%)	2	2	2	2 (1)	2+0 (0)	1 (100%)
	Congenital muscular dystrophy	115 (109)	25 (13–47)	58 (50%)	57 (50%)	16+0 (19+9)	24 (21%)	2	2	2	2 (1)	0+0 (0)	1 (100%)
	Skeletal muscle channelopathy	90 (77)	38 (21–52)	47 (52%)	43 (48%)	16+8 (4+7)	29 (32%)	0	0	0	0	..	0
	Overall panel C	860 (772)	34 (16–57)	483 (56%)	377 (44%)	17+9 (21+1)	220 (26%)	5	5	5	5 (3)	6+10 (13+0)	3 (100%)
Panel D (*FMR1*)													
	Intellectual disability	6731 (5998)	11 (9–15)	4051 (60%)	2680 (40%)	1+1 (3+1)	1536 (23%)	124	27	21	10 (10)	0+1 (0+4)	1 (10%)

Some patients might have been recruited in more than one disease category, and therefore the total number of patients broken down by disease is larger than the total. Ethnicity data are provided in the appendix (p 37). Family history is reported as the absolute number and percentage of patients with positive family history, defined as the presence of at least a first degree or second degree affected relative.

* Information regarding the age of onset was available for only one individual.

† Clinical features of patients with complex intellectual disability tested in panel B are provided in the appendix (p 34).

**Table 3:** Patients in the 100 000 Genomes Project with pathogenic repeat expansions confirmed by PCR, by repeat expansion panel and clinical presentation

	**Family ID**	**Patient ID**	**Sex**	**Age category, years**	**Gene**	**Repeat expansion contribution to clinical features**
**Panel A (*AR, ATN1, ATXN1, ATXN2, ATXN3, ATXN7, C9ORF72, CACNA1A, FMR1, FXN, HTT,* and *TBP*)**						
Hereditary ataxia	1	1	M	1–40	*AR*	Partial
Hereditary ataxia	2	2	F	71–80	*ATN1*	Full
Hereditary ataxia	3	3	F	71–80	*ATN1*	Partial
Hereditary ataxia	4	4	M	41–50	*ATN1*	Full
Hereditary ataxia	5	5	M	31–40	*ATXN2*	Full
Hereditary ataxia	6	6	M	31–40	*ATXN2*	Full
Hereditary ataxia	7	7	M	41–50	*ATXN3*	Full
Hereditary ataxia	8	8	F	61–70	*ATXN7*	Full
Hereditary ataxia	9	9	F	51–60	*CACNA1A*	Full
Hereditary ataxia	10	10	F	51–60	*CACNA1A*	Full
Hereditary ataxia	11	11	F	61–70	*FXN*	Full
Hereditary ataxia	12	12	F	41–50	*FXN*	Full
Hereditary ataxia	12	13	F	41–50	*FXN*	Full
Hereditary ataxia	13	14	F	51–60	*HTT*	Full
Hereditary ataxia	14	15	F	71–80	*HTT*	Partial
Hereditary ataxia	15	16	F	51–60	*HTT*	Full
Hereditary ataxia	16	17	F	61–70	*HTT*	Full
Hereditary ataxia	17	18	F	61–70	*TBP*	Full
Hereditary ataxia	18	19	F	51–60	*TBP*	Full
Hereditary spastic paraplegia	19	20	M	11–20	*ATXN1*	Partial
Hereditary spastic paraplegia	20	21	M	51–60	*FXN*	Full
Hereditary spastic paraplegia	21	22	F	51–60	*HTT*	Full
Early-onset Parkinson's disease	22	23	M	61–70	*ATXN2*	Full
Early-onset Parkinson's disease	23	24	M	31–40	*C9orf72*	Case under review*
Complex parkinsonism	24	25	M	51–60	*ATXN3*	Full
Complex parkinsonism	25	26	F	51–60	*HTT*	Full
Early-onset dementia	26	27	M	51–60	*ATN1*	Full
Early-onset dementia	27	28	F	71–80	*C9orf72*	Full
Early-onset dementia	28	29	M	81–90	*C9orf72*	Full
Early-onset dementia	29	30	M	41–50	*C9orf72*	Full
Amyotrophic lateral sclerosis	30	31	M	51–60	*AR*	Full
Amyotrophic lateral sclerosis	30	32	F	71–80	*AR*	Full
Amyotrophic lateral sclerosis	31	33	M	41–50	*AR*	Full
Amyotrophic lateral sclerosis	32	34	M	51–60	*AR*	Full
Amyotrophic lateral sclerosis	33	35	M	31–40	*ATXN2*	Partial
Amyotrophic lateral sclerosis	34	36	M	71–80	*C9orf72*	Full
Amyotrophic lateral sclerosis	35	37	M	71–80	*C9orf72*	Full
Amyotrophic lateral sclerosis	36	38	F	61–70	*C9orf72*	Full
Charcot-Marie-Tooth disease	37	39	M	61–70	*AR*	Full
Charcot-Marie-Tooth disease	38	40	M	41–50	*AR*	Full
Charcot-Marie-Tooth disease	39	41	M	21–30	*AR*	Full
Charcot-Marie-Tooth disease	40	42	M	21–30	*AR*	Partial
Ultra-rare disorders	41	43	M	31–40	*FXN*	Partial
Ultra-rare disorders	42	44	F	61–70	*HTT*	Full
Ultra-rare disorders	42	45	F	61–70	*HTT*	Full
**Panel B (*ATN1, ATXN1, ATXN2, ATXN3, ATXN7, CACNA1A,* and *HTT*)**						
Early-onset dementia	26	46	M	11–20	*ATN1*	Full
Intellectual disability	4	47	F	11–20	*ATN1*	Full
Intellectual disability	7	48	F	1–10	*ATXN2*	Full
Intellectual disability	43	49	F	1–10	*ATXN7*	Full
Mitochondrial disorders	44	50	F	1–10	*ATXN7*	Full
Mitochondrial disorders	45	51	F	1–10	*HTT*	Full
Early-onset dystonia	46	52	M	11–20	*HTT*	Full
Ultra-rare disorders	47	53	F	1–10	*ATXN7*	No
**Panel C (*DMPK*)**						
Distal myopathies	48	54	F	21–30	*DMPK*	Full
Distal myopathies	48	55	M	41–50	*DMPK*	Full
Congenital myopathy	49	56	M	41–50	*DMPK*	Full
Congenital muscular dystrophy	50	57	F	41–50	*DMPK*	Full
Congenital muscular dystrophy	50	58	F	11–20	*DMPK*	Full
**Panel D (*FMR1*)**						
Intellectual disability	51	59	M	1–10	*FMR1*	Partial
Intellectual disability	52	60	M	1–10	*FMR1*	Full
Intellectual disability	53	61	M	11–20	*FMR1*	Full
Intellectual disability	54	62	M	1–10	*FMR1*	Partial
Intellectual disability	55	63	M	11–20	*FMR1*	Full
Intellectual disability	56	64	M	1–10	*FMR1*	Full
Intellectual disability	57	65	M	1–10	*FMR1*	Full
Intellectual disability	58	66	M	1–10	*FMR1*	Partial
Intellectual disability	59	67	M	1–10	*FMR1*	Full
Intellectual disability	60	68	F	11–20	*FMR1*	Partial

Further details, including additional phenotypic information and repeat size estimates by ExpansionHunter, are provided in the appendix (p 33). The repeat expansion contribution to the patient phenotype was assessed by the local recruiting clinician. M=male. F=female.

* The patient needs further clinical assessment to establish the contribution of the repeat expansion to his clinical features.

Expansions were observed in patients presenting with a wide variety of overlapping clinical presentations tested with panel A (table 3, appendix p 22), including an *ATXN2* repeat expansion in a patient with levodopa-responsive early-onset Parkinson's disease and a history of progressive cerebellar ataxia, and *AR* expansions in four patients clinically diagnosed with Charcot-Marie-Tooth disease, including one with a genetically confirmed demyelinating neuropathy (ie, Charcot-Marie-Tooth disease type 1, patient 42; appendix p 33). A wide range of previous clinical diagnoses were observed in patients with pathogenic repeat expansions. For example, in seven patients with amyotrophic lateral sclerosis or other motor neuron disease, expansions were identified in *AR* (n=4) and *C9orf72* (n=3). In patients with suspected hereditary ataxia, we identified expansions in loci that had not been assessed as part of routine diagnostic workup within the NHS at the time of recruitment, including *ATN1*, *ATXN2*, *ATXN3*, *ATXN7*, *CACNA1A*, *FXN*, *TBP*, and *HTT* (table 3). We also detected repeat expansions in patients with clinical features consistent with alternative repeat expansion disorders, including a *C9orf72* expansion in early-onset and familial Parkinson's disease (patient 24, table 3) and repeat expansions in the reduced penetrance range in *HTT* (38 repeats) in two sisters with movement disorder, dementia, depression, and speech difficulties (patients 44 and 45), underscoring the diagnostic challenge presented by these repeat expansion disorders.

**Figure 3:**
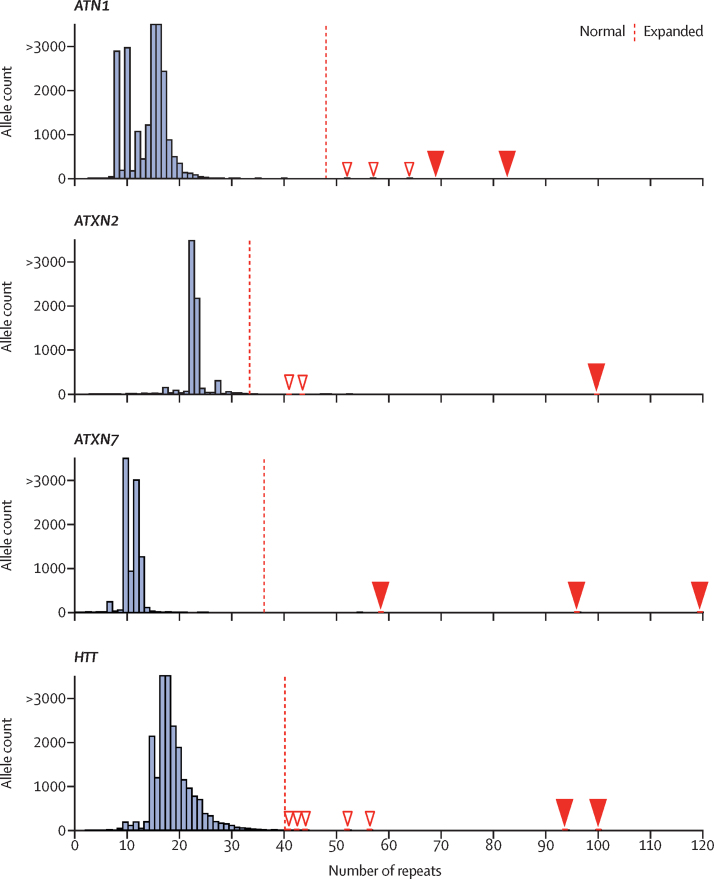
Adult and paediatric patients showing pathogenic expanded repeats Repeat size frequency distribution of genes for which a repeat expansion was detected in paediatric patients (*ATN1*, *ATXN2*, *ATXN7*, and *HTT***)** in 11 631 patients. The number of CAG repeats relative to allele count is shown. The children with large expansions are described in table 3 (*ATN1* in patients 46 and 47; *ATXN2* in patient 48; *ATXN7* in patients 49, 50, and 53; *HTT* in patients 51 and 52). The dashed red line represents the full mutation threshold, above which the number of repeat expansions is considered to be pathogenic for each locus (appendix p 28). White arrowheads indicate pathogenic expansions detected in adults and red arrowheads indicate pathogenic expansions detected in children.

Eight children tested with panel B were found to have large CAG repeat expansions (figure 3), seven of which fully explained the patient's clinical features. Six patients did not have an informative family history and had not been offered repeat expansion testing as part of their clinical assessment at the time of recruitment (patients 48–53; table 3, appendix p 33). Two of these children carried large *HTT* expansions (90–100 CAG repeats). Of note, one child had inherited the repeat from an unaffected parent with no family history of Huntington's disease. Family testing is ongoing, but a reduced penetrance allele has been identified in the extended family, indicating that the repeat had expanded by over 60 repeat units in a single generation (patient 52). At the time of writing, no one in the family showed any signs of Huntington's disease, and genetic counselling and testing are ongoing for the parents. Two children younger than 5 years carried large repeat expansions in *ATXN7* and presented with complex multi-system disease. For one of these children (patient 50), their parent showed gait problems 2 years after enrolment in the 100 000 Genomes Project. Similarly, a girl aged 10 years with intellectual disability was found to have a 99-repeat expansion in *ATXN2*, despite the fact that both parents were designated as unaffected, and a girl aged 18 years with dementia was found to carry a 69-repeat expansion in *ATN1* (appendix p 33).

Five expansions in *DMPK* (panel C) were detected, including in a child and a mother with a clinical diagnosis of muscular dystrophy, in two siblings with suspected distal myopathy, and in an adolescent with congenital myopathy (patients 54–58). *FMR1* expansions (panel D) were detected in nine boys and one girl, and a diagnosis of Fragile X syndrome fully or partially explained the presenting clinical features (patients 59–68).

## Discussion

The diagnosis of repeat expansion disorders is challenging in health care due to heterogeneous and overlapping clinical features and non-specific clinical findings, which can increase in severity with age and in each subsequent generation. Repeat expansion disorders are among the most common causes of inherited neurological diseases.^4^ Nonetheless, patients might be underdiagnosed, either because insufficient genetic testing has been performed or because the causative genetic variants have yet to be discovered. Testing approaches are currently fragmented, and patients might have the incorrect repeat expansion locus tested^29^ or receive a molecular test for a different class of variant due to overlap of clinical features with other neurological genetic disorders.^30^

Whole genome sequencing has been used in multiple settings as a first-line diagnostic test for rare neurological disorders, but has previously been thought to have low ability to detect repeat expansions.^16^ Several tools have been developed to identify repeat expansions from whole genome sequencing in the research setting,^31^ but none of these approaches has been applied to whole genome sequencing data collected from a large number of patients in a single health-care service. We present evidence that an algorithm designed to detect repeat expansions from whole genome sequencing can reliably assess the most common disease-causing repeat expansions and resolve previously genetically undiagnosed cases in a large cohort of patients with neurological disorders. Our results indicate that whole genome sequencing can distinguish between non-expanded and expanded alleles with high sensitivity and specificity across 13 repeat expansion loci (which can be further improved by visual inspection), can accurately calculate the size of alleles smaller than the read length, and might underestimate the size of large expansions in *FMR1*, *DMPK*, *FXN*, and *C9orf72*.

When detection of repeat expansions by whole genome sequencing was assessed against positive and negative results previously obtained at clinical diagnostic genomic laboratories using gold-standard methods, we found a minimum of 97·3% sensitivity and 99·6% specificity. Furthermore, we showed that both the specificity and the sensitivity can be improved by manual curation of the read pileup, enabling detection of false positive results and reclassification of false negative alleles in samples with biallelic expansions. Of the 6731 patients tested for *FMR1* (panel D), 124 calls were predicted to be expanded. We were able to exclude 97 through visual inspection as likely false positives. This indicates that 1 in 54 whole genome sequencing tests would have a *FMR1* call that would need to be visually inspected to discard a potential false positive call. Work is ongoing to improve the ExpansionHunter genotyping method to reduce the number of false positive calls for *FMR1*.

We show that repeat sizing is accurate for repeats smaller than the sequencing read lengths, and therefore that most non-expanded and premutation CAG repeat expansion disorder alleles can be sized accurately. These results are consistent with other studies showing a strong correlation between whole genome sequencing and PCR quantification of repeat lengths smaller than the sequencing read length.^19^, ^25^, ^26^ Whole genome sequencing expansion detection is limited in its sizing of alleles considerably larger than the read length, such as in Fragile X syndrome. We note that all *FMR1* repeats previously classified by PCR as fully expanded (ie, >200 repeats) were classified by whole genome sequencing as premutation (50–200 repeats) in this study. Repeat size estimation for repeats larger than the read length is particularly important for loci in which the length of the repeat correlates with the disease clinical features. This includes *DMPK*, for which small expansions (50–150 repeats) cause mild myotonic dystrophy type 1 and large expansions (>1000 repeats) cause more severe disease, and spinocerebellar ataxia type 36 (*NOP56*), for which expansions larger than 650 repeats are considered pathogenic and repeat sizes of 15–650 are considered intermediate and variants of uncertain significance.

More than 40 repeat expansion loci have been identified; many of these loci have only been identified recently and are now associated with previously unexplained conditions, including cerebellar ataxia with neuropathy and vestibular areflexia syndrome (*RFC1*)^32^ and myoclonic epilepsy (*SAMD12*).^33^ The most common neurological disease-causing repeat expansion loci were selected for our study based on the availability of positive and negative control samples.

The findings presented here suggest that ExpansionHunter should be able to classify non-expanded and expanded alleles accurately at any repeat expansion locus if the non-expanded alleles are smaller than the read length (ie, 150 bp). Although most repeat expansion loci have alleles that are smaller than 150 bp when non-expanded, some loci for which the size of the non-expanded allele is close to 150 bp (eg, *NOTCH2NLC)*^34^ might be more difficult to genotype using this approach. For loci where the expanded repeat is significantly larger than the read length, whole genome sequencing can detect pathogenic expansions (eg, *NOP56,*^35^ *RFC1*^20^, ^32^). Emerging long-read sequencing technologies might offer complementary approaches when genotyping large expansions.^36^

Assessment of repeat expansions using whole genome sequencing in 11 631 undiagnosed patients recruited to the 100 000 Genomes Project yielded 68 patients with explanatory findings. Patients were recruited to the 100 000 Genomes Project after standard-of-care genetic testing; therefore, the proportion of repeat expansions identified in this cohort represents an uplift of the diagnostic yield from standard NHS testing, which includes locus-specific testing for repeat expansion disorders such as *FXN* or *DMPK*. Of note, some diagnoses were not suspected based on the patient's clinical features, including six paediatric patients who had no known family history of a repeat expansion disorder. The mean repeat expansion sizes predicted by whole genome sequencing in paediatric patients described in this study are substantially larger than the mean in adults, consistent with the expectation that larger expansions are associated with earlier and more severe onset, even in children. Further work is needed, but this finding suggests that an age-dependent and repeat size-dependent assessment of pathogenicity might support paediatric diagnosis by reducing the potential hazard of identifying adult-onset risk alleles, leading to unsolicited predictive testing in children.

Our findings enable the establishment of a clinical diagnostic workflow for whole genome sequencing (appendix p 23). We propose that visual inspection is done for all calls classified as expanded to detect false positives, and for biallelic expansions for which only one expanded allele has been detected (eg, *FXN*). We recommend that laboratories use ExpansionHunter to assess for the presence of an expansion without adherence to size estimation, and perform confirmatory PCR testing as a standard component of the testing workflow.

Rare inherited diseases include a wide range of clinical features, making locus-specific genomic testing inefficient, arduous, and expensive. We present evidence that clinical grade whole genome sequencing with the potential to diagnose a range of rare neurological diseases typically presenting with single base, indel, or copy number variants could now be extended to repeat expansions. Because whole genome sequencing provides a single test that can identify the most common repeat expansions, as well as enabling testing of point mutations and copy number variants in genes associated with these conditions simultaneously, it offers the opportunity to identify most patients with these heterogeneous disorders who have not been diagnosed using locus-specific testing. In the era of emerging therapies for these disorders, early detection might become crucial.^37^ These results support implementation of whole genome sequencing for detection of repeat expansions in clinical diagnostic laboratories, an approach that has already been included in the NHS England National Genomic Test Directory,^38^ for investigation of undiagnosed rare neurological disease.

## Data sharing

De-identified clinical information included in this analysis is provided in the appendix. Additional information on data access can be found at https://www.genomicsengland.co.uk/about-gecip/for-gecip-members/data-and-data-access. For the 150 patient samples sequenced by ICSL, de-identified bam files for the regions around the tested repeats will be made available upon request. Requests should be addressed to Ryan J Taft (rtaft@illumina.com). Data use will be restricted to Illumina-approved research questions performed under an institutional review board-approved research protocol. Data will be available for request for 2 years.

## Declaration of interests

Genomics England is a company wholly owned by the UK Department of Health and Social Care and was created in 2013 to introduce whole genome sequencing into health care in conjunction with NHS England. All authors affiliated with Genomics England (KI, DP, ERAT, LCD, DK, KRS, TF, RHS, AR, MJC, and AT) are, or were, salaried by or seconded to Genomics England. RJT, MAE, ED, and RTH are employees and shareholders of Illumina. PFC is in receipt of a grant from the Wellcome Trust Medical Research Council (MRC). All other named authors declare no competing interests. Declarations of interests for members of the WGS for Neurological Diseases Group are provided in the appendix (p 14).
