# Supplementary material for: Whole genome sequencing for the diagnosis of neurological repeat expansion disorders in the UK: a retrospective diagnostic accuracy and prospective clinical validation study
Source: Lancet Neurol. 2022 Mar;21(3):234–45. doi: 10.1016/S1474-4422(21)00462-2 (PMC8850201; doi:10.1016/S1474-4422(21)00462-2)
Supplement: Supplementary appendix [file mmc1.pdf]

THE LANCET
Neurology

Supplementary appendix

This appendix formed part of the original submission and has been peer reviewed. We post it as supplied by the authors.

Supplement to: Ibañez K, Polke J, Hagelstrom RT, et al. Whole genome sequencing for the diagnosis of neurological repeat expansion disorders in the UK: a retrospective diagnostic accuracy and prospective clinical validation study. *Lancet Neurol* 2022; **21:** 234–45.

SUPPLEMENTARY METHODS

Repeat expansion performance datasets

Whole genome sequencing and locus-specific PCR data from patients historically tested for repeat expansion diseases was obtained from two sources: patients from Genomics England (GE) in the 100,000 Genomes Project and from Illumina Clinical Services Laboratory (ICSL). For patients from GE locus-specific PCR tests were obtained from patients that had been screened for repeat expansion prior to recruitment or tested during the pilot phase of the 100,000 Genomes Project by the Neurogenetics Laboratory at the UCLH National Hospital for Neurology and Neurosurgery. The GE dataset consisted of 254 patient samples corresponding to 634 locus-specific PCR tests. For each patient both alleles at each locus were assessed, with the exception of loci on chromosome X in males, yielding 1,172 non-expanded alleles, 26 premutation alleles and 35 expanded alleles. ICSL samples were collected from the Genetics Laboratory at the Cambridge University Hospitals NHS Foundation Trust and included 150 patient samples corresponding to 159 locus-specific PCR tests comprising 149 non-expanded alleles and 160 expanded alleles (Table S1).

Repeat expansion loci analysed

Eleven repeats associated with ataxia and late-onset neurodegenerative disorders were tested, including all exonic CAG repeat disorders - Huntington disease (*HTT*; #143100), spinal and bulbar muscular atrophy of Kennedy (*AR*; #313200), dentatorubral-pallidoluysian atrophy (*ATN1*; #125370), spinocerebellar ataxia 1 (*ATXN1*; #164400), spinocerebellar ataxia 2 (*ATXN2*; #183090), Machado-Joseph disease (*ATXN3*; #109150), spinocerebellar ataxia 7 (*ATXN7*; #164500), spinocerebellar ataxia 6 (*CACNA1A;* #183086), and spinocerebellar ataxia 17 (*TBP*; #607136); and two known intronic repeat disorders - frontotemporal dementia and/or amyotrophic lateral sclerosis 1 (*C9orf72*; #105550) and Friedreich ataxia (*FXN*; #229300). Further, we investigated performance for Fragile X syndrome (*FMR1*; #300624) and Myotonic dystrophy 1 (*DMPK*; *#160900*). Genomic coordinates defined for the region of each locus used by ExpansionHunter versions ExpansionHunterv2.5.5 and ExpansionHunterv3.1.2 can be found in Table S13 and Table S14 respectively.

Statistical analysis

The statistics formulas used to assess the repeat expansion performance dataset have been taken from https://www.medcalc.org/calc/diagnostic_test.php. Considering TN = True Negative; FP = False Positive; TP = True Positive; FN = False negative:

$$sensitivity = \frac{TP}{(TP + FN)}$$ Equation (1)

$$\boldsymbol{specificity = \frac{TN}{(TN + FP)}}$$ Equation (2)

$$accuracy = sensitivity \times prevalence + specificity\ x\ (1 - prevalence)$$ Equation (3)

$$positive\ predictive\ value = \frac{sensitivity\ x\ prevalence}{sensitivity\ x\ prevalence + (1 - specificity)\ x\ (1 - prevalence)}$$ Equation (4)

$$\boldsymbol{negative\ predictive\ value = \frac{specificity\ x\ (1 - prevalence)}{(1 - sensitivity)\ x\ prevalence + specificity\ x\ (1 - prevalence)}}$$ Equation (5)

The prevalence of repeat expansions in the population was estimated using the disease prevalences in Table S5. We estimate that the probability that someone has of the thirteen repeat expansions assessed in this study is roughly 1:2700. For this calculation we used a prevalence of 0.5:100,000 for any of the diseases that are listed as having a prevalence of <1:100,000**.**

ExpansionHunter code examples

ExpansionHunter requires an indexed BAM or CRAM file containing aligned reads from a PCR-free whole genome sequencing sample, the reference FASTA file with a reference genome assembly used when aligning the BAM/CRAM file, and a variant catalog file:

```
ExpansionHunter --reads <BAM/CRAM file with aligned reads> \
    --reference <FASTA file with reference genome> \
  --variant-catalog <JSON file specifying variants to genotype> \
  --sex <arg> Specifies sex of the sample; can be either male or female \
  --output-prefix <Prefix for the output files>
```

The \`sex\` argument is optional and it only affects repeats on sex chromosome (i.e. *AR* and *FMR1* in this analysis). From ExpansionHunter v3 on the variant catalog is a single JSON file specifying

Each ExpansionHunter release includes a variant-catalog for different assemblies (https://github.com/Illumina/ExpansionHunter/releases), and this should match with the reference used when aligning the genomes. It specifies reference coordinates and structure of each locus that the program will analyse.

An example of the format of the specification for a gene:

```
{
    "LocusId": "ATN1",
    "LocusStructure": "(CAG)*",
    "ReferenceRegion": "chr12:6936716-6936773",
    "VariantType": "Repeat"
}
```

The identifier of this locus is *ATN1* (field \`LocusId\`). The regular expression $(CAG)*$means that it consists of zero or more repetitions of the \`CAG\` repeat unit (field \`LocusStructure\`). The reference coordinates of this repeat are chr12:6936716-6936773 (field \`ReferenceRegion\`). And the \`VariantType\` field specifies that it is an ordinary RE, meaning that it is expected the genome to contain multiple long repeats (whose size is close to fragment length or longer) with this repeat unit (\`CAG\`). For more information, please visit https://github.com/Illumina/ExpansionHunter/blob/master/docs/04_VariantCatalogFiles.md.

PCR analysis

Genomic DNA was isolated from peripheral blood leukocytes following standard protocols. PCR-based and Southern blot analysis of repeat expansion loci was performed by the Neurogenetics Laboratory at the UCLH National Hospital for Neurology and Neurosurgery for all Genomics England samples. The Genetics Laboratory, Cambridge University Hospitals NHS Foundation Trust assessed all ICSL sequenced samples as part of routine clinical assessment.

Neurogenetics Laboratory at the National Hospital for Neurology and Neurosurgery

All PCR assays and Southern blots followed accredited diagnostic protocols established in the laboratory. Allele sizing by fluorescent tethered repeat-primed PCR was previously validated against Sanger-sequenced controls, and control samples of a known repeat expansion size were included in each assessment.

SCA1-7 (*ATXN1, ATXN2, ATXN3, CACNA1A, ATXN7*): Fluorescent tethered repeat-primed PCR (RP-PCR) was performed for each locus using the primers listed in Table S2, with PCR conditions modified from Cagnoli et al.[1] Large SCA7 expansion that could not be sized by tethered RPPCR were amplified with flanking primers (Table S2) and assessed by electrophoresis on a 2% agarose gel against a 1 kb size standard (Promega).

SCA17 (*TBP*): A fluorescent flanking PCR was performed using the primers listed in Table S2, based on Nethsinghe et al.[2]

HD (*HTT*): Two fluorescent PCRs were performed, both based on the method published by Warner et al.[3] The first PCR was a tethered RP-PCR interrogating the CAG repeat size. The second PCR was a flanking PCR also capturing an adjacent highly polymorphic 'CCG' repeat. See Table S2 for primer sequences.

FRDA (*FXN*): A three-primer fluorescent RP-PCR assay was used following the protocol established by Warner et al,[4] with the 3rd 'non-genomic' primer complementary to the tail of the repeat-binding primer, and a 'long PCR' with flanking primers that was analysed both fluorescently and on 2% agarose gel to approximate the size of the pathogenic expansions.[5] See Table S2 for primer sequences.

SBMA (*AR*): A fluorescent flanking PCR was performed using published primers[6] listed in Table S2.

DRPLA (*ATN1*): A tethered fluorescent RP-PCR was used, using the primers listed in Table S2 and under standard PCR conditions.

FTD/ALS (*C9orf72*): Two fluorescent RP-PCRs were performed, amplifying opposite ends of the repeat, utilising published primers. RP-PCR1 is derived from Renton et al[7] and RP-PCR2 (a tethered RP-PCR) from DeJesus-Hernandez et al.[8] These were complemented by a flanking PCR, using primers from DeJesus-Hernandez et al.[8] See Table S2 for sequences. Expansions detected by RP-PCR were confirmed and sized by Southern blotting using a 1 kb single copy probe as previously described[9] but using BsU36I restriction enzyme digests that generate a 6.2 kb band for unexpanded alleles, rather than the EcoRI digest used previously that generates an 8 kb band described in the original protocol.

Whole genome sequencing Repeat Expansion genotyping and visual inspection

STR genotyping from whole genome sequencing was performed using ExpansionHunter.[10,11] In brief, ExpansionHunter (ExpansionHunter) aligns reads to a modified segment of the reference genome that can accommodate STRs of any length. The algorithm employs either an ad hoc[10] or graph-based approach.[11] When the STR is shorter than the whole genome sequencing read length (e.g. 150 bp) the genotype is identified from the realigned reads. When the STR is longer than the read length, its size is estimated from the number of reads that align to the locus plus those that are entirely composed of repeat sequences (i.e. in-repeat reads). We used two versions of ExpansionHunter, (ExpansionHunterv3.1.2 and ExpansionHunterv2.5.5) for testing diagnostic accuracy of whole genome sequencing to detect repeat expansions (Table S4). The diagnostic accuracy was not affected by ExpansionHunter version (Table S6 and Table S16). Read-coverage of the loci analysed are shown in the `Locus coverage` column in Table S4.

Visual inspection

Visual inspection of complex whole genome sequencing variant calls, using tools like Integrated Genome Viewer, IGV,[12] is standard practice in most clinical laboratories. Because repeat expansions can include a significant amount of inserted sequence relative to the reference genome, these common visualisations are not adequate for repeat expansion investigation. To address this gap we used a tool that creates a static visualisation of the read pileup against an alternative reference constructed from the putative expanded allele identified by ExpansionHunter (Figure S1A and S1B). This tool can be downloaded from https://github.com/Illumina/GraphAlignmentViewer and makes it possible to inspect the evidence used by ExpansionHunter to make a genotype call and identify putative false positives. Additionally, it enables rapid visual inspection of data quality by representing low quality (i.e. <Q20) bases as lower case, facilitating identification of genomic regions or samples that may be impacted by poor data quality. From the visualisation, a user is able to identify and interrogate sequencing reads that align to the region allowing for an additional assessment of the ExpansionHunter calls, analogous to how IGV is used to visually confirm SNP calls.

Interpreting pileup plots

To interpret a pileup plot, first it is important to understand how ExpansionHunter estimates repeat sizes from whole genome sequencing at a given locus. ExpansionHunter scans the whole genome sequencing BAM file to identify reads that (1) either fully span the repeat (spanning reads); or (2) include the repeat and the flanking sequence on one side of the repeat (flanking reads); or (3) are fully contained in the repeat ("in-repeat" reads, IRR). It does this by creating a dynamic graph reference genome where the repeat is represented by a loop in the graph and the reads are realigned to this dynamic reference.[10,11] If the repeat is shorter than the read length of the sequence data (e.g. an expansion in *CACNA1A*), there should be some spanning reads and an exact size is identified. To estimate the length of a repeat longer than the read length (e.g *C9orf72* or *FXN*), IRRs are identified and counted. When the repeat length is close to the read length, the size of the repeat is approximated from the flanking reads that partially overlap the repeat and one of the repeat flanks. If the repeat is longer than the read length, its size is estimated from IRR. In-repeat reads anchored by their mate to the repeat region are used to estimate the size of the repeat up to the fragment length.

The pileup plot is a graphic representation of the sequencing reads that align to the repeat region of interest. Within a pileup graph, the reads supporting each genotype are grouped based on (i) the type of reads: spanning, flanking or IRR; (ii) the repeat length supported by each group. Furthermore, sequencing quality of each base of the read is represented by upper case letters for high quality (>Q20 ; error rate less than 1%) bases or lower case base letters for low quality (<Q20 ; error rate >1%) bases.

In our experience, genotyping errors may occur if reads are classified incorrectly due low quality data or mosaicism. The sequencing reads represented in the pileup plot can be inspected and interrogated to confirm the repeat length predicted by ExpansionHunter. Figure S2 shows three examples of different pileup plots that support the genotype predicted by the correspondent ExpansionHunter call:

- Case A: A monoallelic expansion smaller than the read length where 9 spanning reads support the shorter repeat (22 repeats in green box) and two spanning reads support the longer repeat (40 repeats in green box). In addition to the spanning reads there are multiple flanking reads that have up to 39 repeats and provide further evidence of the expansion.
- Case B: A monoallelic expansion larger than the read-length where 14 spanning reads support the shorter allele (2 repeats) and ~26 IRRs provide strong evidence for the expansion.
- Case C: A biallelic expansion larger than the read length where there are no spanning reads for a short allele and ~44 IRRs supporting long alleles. We would expect fewer IRRs if there was only a single expansion - note that there are roughly twice as many IRRs in this example as observed for the monoallelic expansion of case B.

Repeat sizing

A total of 509 PCR tests were analysed corresponding to 945 alleles (marked as \`Yes\` in \`repeat_sizing_test_any\` column in Table S4). Table S4 provides the allele sizes from PCR alone and the repeat-size estimates of both ExpansionHunter versions.
In this analysis, we assumed that the PCR call is correct. In order to analyse the correlation between PCR and whole genome sequencing repeat-size estimates, repeats for which PCR exact lengths that were smaller than the read-length (i.e. 150bp) were available for at least one allele were taken into account (n=902). The columns \`included_in_concordance_test_PCR_smaller_read-length_aX\` in Table S4 (\`Yes\`/\`No\`) define whether each allele (a1, a2) is included for this analysis or not. The concordance overall and and by locus is computed from Table S4, where \`concordance_PCR_length_ExpansionHunterv312_length_1_repeat_error_aX\` columns indicate whether PCR and ExpansionHunter sizes are in agreement (\`NA\` if the allele is not included).

Inclusion criteria for testing 100,000 Genomes Project patients using the whole genome sequencing pipeline to detect repeat expansions

Patients were tested using four different virtual repeat expansion panels (A-D): Panel A) 'Neurodegenerative': *AR, ATN1, ATXN1, ATXN2, ATXN3, ATXN7, CACNA1A, C9orf72, FMR1, FXN, HTT*, or *TBP*; B) 'Complex Intellectual disability': *HTT, ATN1, ATXN2, ATXN3, CACNA1A,* and *ATXN7*; C) `neuromuscular': *DMPK*; D) 'Intellectual disability': *FMR1*. Patients were tested with one or more virtual panel(s) according to their phenotype. To filter out putatively expanded alleles, full-mutations cutoffs were used except for *FMR1* given that whole genome sequencing cannot distinguish between full and permutations, and *HTT* were 38 was used as these alleles can have reduced penetrance. Full-mutation and premutation cutoffs are listed in Table S5.

Panel A included patients recruited under any of the following diseases: amyotrophic lateral sclerosis or motor neuron disease, Charcot-Marie-Tooth disease, early onset dementia, early onset dystonia, complex parkinsonism, hereditary ataxia, hereditary spastic paraplegia, early onset and familial Parkinson's disease. For all these diseases only adults (i.e. patients equal or older than 18 years old in 2020) were selected except for hereditary ataxia, where children were also included. Furthermore, patients recruited under ultra-rare undescribed monogenic disorders (i.e. patients that did not fit clinically a specific eligible disease) and whose HPO terms were suggestive of ataxia or a neurodegenerative disorder were also included.

Panel B included paediatric patients that whose HPO terms included "intellectual disability" plus or or more of: "seizures"/ "epilepsy"; "hypotonia"/"muscle weakness/myopathy", "ataxia", "spasticity"/"pyramidal signs", "white matter abnormalities"/"leukodystrophy", "optic atrophy".

Panel C included patients recruited under any of the following diseases: congenital myopathy, distal myopathies, congenital muscular dystrophy, skeletal muscle channelopathy.

Finally, Panel D included children (i.e. patients younger than 18 years old in 2020) whose HPO terms included "Intellectual disability".

For all these four panels the total number of repeat expansions detected before and after visual inspection is presented in Table S9.

100,000 Genomes Project eligibility statements

Patients were recruited to the 100,000 Genomes Project according to the eligibility criteria for conditions approved within the Genomics England Rare Diseases Programme. Eligibility criteria for the conditions analysed in this study are listed below. For further information, please refer to "Rare Disease Conditions Eligibility Criteria - 100,000 Genomes Project".[13]

CHARCOT-MARIE-TOOTH DISEASE

Inclusion criteria

- Unexplained peripheral neuropathy affecting motor, sensory or autonomic nerves progressing over >2 years +/- additional neurological signs.

Exclusion criteria

- History of trauma.
- Known acquired metabolic, vascular, inflammatory or immunological cause - History of alcohol excess.
- Evidence of malignancy.
- ENG/EMG suggest acquired pathology.

Prior genetic testing guidance

- Results should have been reviewed for all genetic tests undertaken, including disease-relevant genes in exome sequencing data. The patient is not eligible if they have a molecular diagnosis for their condition.

- Genetic testing should continue according to routine local practice for this phenotype regardless of recruitment to the project; results of these tests must be submitted via the ‘Genetic investigations’ section of the data capture tool to allow comparison of whole genome sequencing with current standard testing.
- PLEASE NOTE: The sensitivity of whole genome sequencing compared to current diagnostic genetic testing has not yet been established. It is therefore important that tests which are clinically indicated under local standard practice continue to be carried out.

Prior genetic testing genes

- Testing for the chromosome 17p11.2 duplication is strongly recommended PRIOR TO RECRUITMENT as this may not be reliably detected by whole genome sequencing using current analysis techniques; other tests below should be considered where this is in line with current local practice including:
- *PMP22* point mutations, *GJB1, MPZ, MFN2* (*MFN2* axonal only)

EARLY ONSET DEMENTIA

Early onset dementia inclusion criteria

- Progressive cognitive deterioration with change in memory, vision, behaviour or language with functional impairment
- Age at onset <60 years OR
- Later onset with family history of dementia of the same type in a first or second degree relative
- Patients with severe or syndromic disease should be recruited according to standard guidance, typically as trios. Disease status of apparently unaffected patients should be determined according to standard clinical practice to detect cryptic disease. In other cases, unaffected patients should not be recruited. Recruitment in such families should favour multiplex families over single isolated cases. These singleton recruits will not contribute to the overall singleton monitoring metrics applied to GMCs.

Early onset dementia exclusion criteria

- Identified underlying cause, e.g. structural brain lesion. NB in uncertain cases with anxiety/depression brain atrophy on imaging, CSF findings or EEG abnormalities should be available to support the diagnosis of a primary degenerative syndrome

Prior genetic testing guidance

- Results should have been reviewed for all genetic tests undertaken, including disease-relevant genes in exome sequencing data. The patient is not eligible if they have a molecular diagnosis for their condition.
- Genetic testing should continue according to routine local practice for this phenotype regardless of recruitment to the project; results of these tests must be submitted via the ‘Genetic investigations’ section of the data capture tool to allow comparison of whole genome sequencing with current standard testing.
- PLEASE NOTE: The sensitivity of whole genome sequencing compared to current diagnostic genetic testing has not yet been established. It is therefore important that tests which are clinically indicated under local standard practice continue to be carried out.

Early onset Dementia prior genetic testing genes

- Testing of the following genes should be carried out PRIOR TO RECRUITMENT where this is in line with current local practice:
  - Clinical syndrome Alzheimer disease: *PSEN1, APP*
  - Clinical syndrome FTLD: *MAPT, C9ORF72, GRN*
  - Clinical syndrome Prion disease: *PRNP*

- PLEASE NOTE: The sensitivity of whole genome sequencing compared to current diagnostic genetic testing has not yet been established. It is therefore important that tests which are clinically indicated under local standard practice continue to be carried out.

EARLY ONSET DYSTONIA

Early onset dystonia inclusion criteria

- Dystonia affecting any body part, usually spreading to involve multiple body regions (e.g. multifocal, segmental, generalised)
- Age at onset <31 years or later onset with family history of early onset dystonia
- May be paroxysmal/episodic dystonia
- May be associated with myoclonus as in myoclonic dystonia
- This disease category includes dopa responsive dystonia.

Early onset dystonia exclusion criteria

- Underlying cause for clinical syndrome identified, e.g. cerebral palsy, structural brain lesion, Wilson disease, psychogenic dystonia

Prior genetic testing guidance

- Results should have been reviewed for all genetic tests undertaken, including disease-relevant genes in exome sequencing data. The patient is not eligible if they have a molecular diagnosis for their condition.
- Genetic testing should continue according to routine local practice for this phenotype regardless of recruitment to the project; results of these tests must be submitted via the 'Genetic investigations' section of the data capture tool to allow comparison of whole genome sequencing with current standard testing.
- PLEASE NOTE: The sensitivity of whole genome sequencing compared to current diagnostic genetic testing has not yet been established. It is therefore important that tests which are clinically indicated under local standard practice continue to be carried out.

Early onset dystonia prior genetic testing genes

- Testing of the following genes should be carried out PRIOR TO RECRUITMENT where this is in line with current local practice:
    - *TOR1A*

COMPLEX PARKINSONISM (INCLUDES PALLIDO-PYRAMIDAL SYNDROMES)

Complex Parkinsonism inclusion criteria

- Progressive motor syndrome with parkinsonism (bradykinesia with one of tremor, gait disorder, stiffness)
- Additional features may include spasticity, gaze palsy, early dementia, early bulbar failure, dyspraxia, ataxia, postural hypotension, cortical sensory loss, brain iron accumulation on MRI brain
- Aat onset <= 45 years or later onset with family history of similar condition in other family members
- Patients with severe or syndromic disease should be recruited according to standard guidance, typically as trios. Disease status of apparently unaffected patients should be determined according to standard clinical practice to detect cryptic disease. In other cases, unaffected patients should not be recruited. Recruitment in such families should favour multiplex families over single isolated cases. These singleton recruits will not contribute to the overall singleton monitoring metrics applied to GMCs.

Complex Parkinsonism exclusion criteria

- Underlying cause not identified, e.g. structural brain lesion, Wilson disease

Prior genetic testing guidance

- Results should have been reviewed for all genetic tests undertaken, including disease-relevant genes in exome sequencing data. The patient is not eligible if they have a molecular diagnosis for their condition.
- Genetic testing should continue according to routine local practice for this phenotype regardless of recruitment to the project; results of these tests must be submitted via the 'Genetic investigations' section of the data capture tool to allow comparison of whole genome sequencing with current standard testing.
- PLEASE NOTE: The sensitivity of whole genome sequencing compared to current diagnostic genetic testing has not yet been established. It is therefore important that tests which are clinically indicated under local standard practice continue to be carried out.

Complex Parkinsonism prior genetic testing genes

- Testing of the following genes should be carried out PRIOR TO RECRUITMENT where this is in line with current local practice:
- *C9ORF72, GRN, MAPT* in cases with a clinical presentation suggestive of cortico-basal/PSP syndrome

HEREDITARY ATAXIA

Hereditary ataxia inclusion criteria

- Unexplained cerebellar ataxia progressing over >2 years +/- spasticity, peripheral neuropathy, or bulbar dysfunction.
- Patients with syndromic disease or disease onset <30 years should be recruited according to standard guidance, typically as trios. Disease status of apparently unaffected patients should be determined according to standard clinical practice to detect cryptic disease.
- In other cases, unaffected patients should not be recruited. Recruitment in such families should favour multiplex families over single isolated cases. These singleton recruits will not contribute to the overall singleton monitoring metrics applied to GMCs.

Hereditary ataxia exclusion criteria

- No structural or inflammatory (MS-like) lesions on brain MRI. - No history of alcohol excess.

- Normal thyroid function.
- No evidence of malignancy.

Prior genetic testing guidance

- Results should have been reviewed for all genetic tests undertaken, including disease-relevant genes in exome sequencing data. The patient is not eligible if they have a molecular diagnosis for their condition.
- Genetic testing should continue according to routine local practice for this phenotype regardless of recruitment to the project; results of these tests must be submitted via the ‘Genetic investigations’ section of the data capture tool to allow comparison of whole genome sequencing with current standard testing.
- PLEASE NOTE: The sensitivity of whole genome sequencing compared to current diagnostic genetic testing has not yet been established. It is therefore important that tests which are clinically indicated under local standard practice continue to be carried out.

Hereditary ataxia prior genetic testing genes

- Testing for genes which are affected by trinucleotide repeats is strongly recommended PRIOR TO RECRUITMENT as these will not be reliably detected by whole genome sequencing using current analysis techniques including: common trinucleotide repeat disorders excluded (*ATXN1, ATXN2, ATXN3, CACNA1A, ATXN7, TBP, ATN1, FXN* (only recessive history), *FMR1*)

HEREDITARY SPASTIC PARAPLEGIA

Hereditary spastic paraplegia inclusion criteria

- Unexplained spastic paraplegia progressing over >2 years +/-, peripheral neuropathy, or ataxia.
- Patients with syndromic disease or disease onset <30 years should be recruited according to standard guidance, typically as trios. Disease status of apparently unaffected patients should be determined according to standard clinical practice to detect cryptic disease.
- In other cases, unaffected patients should not be recruited. Recruitment in such families should favour multiplex families over single isolated cases. These singleton recruits will not contribute to the overall singleton monitoring metrics applied to GMCs.

Hereditary spastic paraplegia exclusion criteria

- No structural or inflammatory (MS-like) lesions on brain MRI.

Prior genetic testing guidance

- Results should have been reviewed for all genetic tests undertaken, including disease-relevant genes in exome sequencing data. The patient is not eligible if they have a molecular diagnosis for their condition.
- Genetic testing should continue according to routine local practice for this phenotype regardless of recruitment to the project; results of these tests must be submitted via the ‘Genetic investigations’ section of the data capture tool to allow comparison of whole genome sequencing with current standard testing.
- PLEASE NOTE: The sensitivity of whole genome sequencing compared to current diagnostic genetic testing has not yet been established. It is therefore important that tests which are clinically indicated under local standard practice continue to be carried out.

Hereditary spastic paraplegia prior genetic testing genes

- Testing of the following genes should be carried out PRIOR TO RECRUITMENT where this is in line with current local practice:
    - *SPAST, ATL1*
    - Normal very long chain fatty acid studies

EARLY ONSET AND FAMILIAL PARKINSON'S DISEASE

Early onset and familial Parkinson's Disease inclusion criteria

- Early onset (<= 45 years of age) or history of other family member with Parkinson’s Disease
- Bradykinesia plus at least one of rigidity, rest tremor and gait disturbance - May have concurrent dystonia (common in early onset PD)
- May have positive family history or consanguinity
- If complex features, e.g. spasticity, early dementia, gaze palsy, Neurodegeneration with Brain Iron Accumulation, please recruit to Complex Parkinsonism
- May develop Lewy Body/PD type dementia
- Patients with severe or syndromic disease should be recruited according to standard guidance, typically as trios. Disease status of apparently unaffected patients should be determined according to standard clinical practice to detect cryptic disease. In other cases, unaffected patients should not be recruited. Recruitment in

such families should favour multiplex families over single isolated cases. These singleton recruits will not contribute to the overall singleton monitoring metrics applied to GMCs.

Early onset and familial Parkinson's Disease exclusion criteria

- Underlying cause for clinical syndrome identified, e.g. cerebral palsy, dopa-responsive dystonia, structural brain lesion, Wilson disease, psychogenic dystonia

Prior genetic testing guidance

- Results should have been reviewed for all genetic tests undertaken, including disease-relevant genes in exome sequencing data. The patient is not eligible if they have a molecular diagnosis for their condition.
- Genetic testing should continue according to routine local practice for this phenotype regardless of recruitment to the project; results of these tests must be submitted via the 'Genetic investigations' section of the data capture tool to allow comparison of whole genome sequencing with current standard testing.
- PLEASE NOTE: The sensitivity of whole genome sequencing compared to current diagnostic genetic testing has not yet been established. It is therefore important that tests which are clinically indicated under local standard practice continue to be carried out.

AMYOTROPHIC LATERAL SCLEROSIS OR MOTOR NEURON DISEASE

Amyotrophic lateral sclerosis or motor neuron disease inclusion criteria

- Progressive upper and/or lower motor neuron disease degeneration with clinical features of amyotrophy, spasticity, bulbar/pseudo-bulbar involvement
- EMG/NCS consistent with MND
- Positive family history of other affected family members with ALS or with FTD/ALS like phenotype or disease onset below 40 years.
- Patients with severe or syndromic disease should be recruited according to standard guidance, typically as trios. Disease status of apparently unaffected patients should be determined according to standard clinical practice to detect cryptic disease. In other cases, unaffected patients should not be recruited. Recruitment in such families should favour multiplex families over single isolated cases. These singleton recruits will not contribute to the overall singleton monitoring metrics applied to GMCs.

Amyotrophic lateral sclerosis or motor neuron disease exclusion criteria

- Identified underlying cause for clinical syndrome e.g. multi-focal motor neuropathy, lymphoma

Prior genetic testing guidance

- Results should have been reviewed for all genetic tests undertaken, including disease-relevant genes in exome sequencing data. The patient is not eligible if they have a molecular diagnosis for their condition.
- Genetic testing should continue according to routine local practice for this phenotype regardless of recruitment to the project; results of these tests must be submitted via the 'Genetic investigations' section of the data capture tool to allow comparison of whole genome sequencing with current standard testing.
- PLEASE NOTE: The sensitivity of whole genome sequencing compared to current diagnostic genetic testing has not yet been established. It is therefore important that tests which are clinically indicated under local standard practice continue to be carried out.

Amyotrophic lateral sclerosis or motor neuron disease prior genetic testing genes

- Testing of the following genes should be carried out PRIOR TO RECRUITMENT where this is in line with current local practice: *C9ORF72, SOD1*

INTELLECTUAL DISABILITY

Intellectual disability inclusion criteria

- Moderate to Severe/ Profound ID disproportionate to parental IQ unless the family history is consistent with an X- linked disorder
- Congenital onset
- Developmental Delay
- +/- clinical features suggestive of a specific syndrome - Metabolic causes have been excluded

Intellectual disability exclusion criteria

- Antenatal history suggestive of non-genetic cause
- Proven congenital or neonatal infections
- Known genetic cause already identified
- Microarray analysis abnormal and clearly pathogenic

Prior genetic testing guidance

- Results should have been reviewed for all genetic tests undertaken, including disease-relevant genes in exome sequencing data. The patient is not eligible if they have a molecular diagnosis for their condition.
- Genetic testing should continue according to routine local practice for this phenotype regardless of recruitment to the project; results of these tests must be submitted via the 'Genetic investigations' section of the data capture tool to allow comparison of whole genome sequencing with current standard testing.

Intellectual disability prior genetic testing genes

- Testing of the following genes should be carried out PRIOR TO RECRUITMENT where this is in line with current local practice:
- For syndromes where the cause of disease is 1-2 genes these need to be excluded before Genomics England recruitment, e.g. for Kabuki syndrome, *MLL2* (*KMT2D*), and *KDM6A* should have been tested

CONGENITAL MUSCULAR DYSTROPHY

Congenital muscular dystrophy inclusion criteria

- Muscle weakness with onset in infancy or early childhood AND
- elevated creatine kinases or muscle biopsy with dystrophic changes - Availability of CK and muscle biopsy results
- Dystrophic changes on muscle biops
- Congenital muscular dystrophy exclusion criteria

Prior genetic testing guidance

- Results should have been reviewed for all genetic tests undertaken, including disease-relevant genes in exome sequencing data. The patient is not eligible if they have a molecular diagnosis for their condition.
- Genetic testing should continue according to routine local practice for this phenotype regardless of recruitment to the project; results of these tests must be submitted via the 'Genetic investigations' section of the data capture tool to allow comparison of whole genome sequencing with current standard testing.

CONGENITAL MYOPATHY

Relevant diseases:

- Congenital myopathy

Congenital myopathy inclusion criteria

- Muscle weakness
- one or more of the following histopathological features
- type 1 predominance or uniformity - congenital fibre type disproportion - central cores
- Multi-minicores
- nemaline rods - central nuclei
- Availability of CK, muscle CT/MR imaging, muscle biopsy and neurophysiological studies

Congenital myopathy exclusion criteria

- Absence of muscle weakness
- CK more than 5x normal
- dystrophic features on muscle biopsy

Prior genetic testing guidance

- Results should have been reviewed for all genetic tests undertaken, including disease-relevant genes in exome sequencing data. The patient is not eligible if they have a molecular diagnosis for their condition.
- Genetic testing should continue according to routine local practice for this phenotype regardless of recruitment to the project; results of these tests must be submitted via the 'Genetic investigations' section of the data capture tool to allow comparison of whole genome sequencing with current standard testing

DISTAL MYOPATHIES

Distal myopathies inclusion criteria

- Unexplained predominantly distal muscle weakness, onset at any age - Acquired myopathies excluded by relevant clinical investigations
- Serum creatine kinase (CK) assessment
- Muscle Biopsy with immunohistochemistry (IH)
- Neurophysiology performed
- Muscle MRI (optional)

- Dried blood spot test for Pompe disease performed

Distal myopathies exclusion criteria

NA

Prior genetic testing guidance

- Results should have been reviewed for all genetic tests undertaken, including disease-relevant genes in exome sequencing data. The patient is not eligible if they have a molecular diagnosis for their condition.
- Genetic testing should continue according to routine local practice for this phenotype regardless of recruitment to the project; results of these tests must be submitted via the 'Genetic investigations' section of the data capture tool to allow comparison of whole genome sequencing with current standard testing.

Distal myopathies prior genetic testing genes

Testing of the following genes should be carried out PRIOR TO RECRUITMENT where this is in line with current local practice:

- *DMD* analysis by MLPA or equivalent
- *DUX1* and *DMPK* exclusion by conventional genetic testing
- Exclusion by genetic testing of any gene indicated by IH.
- In the presence of evidence of myofibrillar myopathy on muscle biopsy IH exclusion of *LDB3, MYOT, DES, CRYAB* by sequencing

SKELETAL MUSCLE CHANNELOPATHIES

Skeletal Muscle Channelopathies inclusion criteria

- Episodic flaccid paralysis or weakness and/or myotonia
- May develop progressive, usually proximal, weakness
- Electrophysiology including long and short exercise testing - Intra-attack potassium documented whenever possible
- Normal renal function and thyroid function

Skeletal Muscle Channelopathies exclusion criteria

- Primary renal or endocrine problem that may be causative - Associated loss of consciousness with attacks

Prior genetic testing guidance

- Results should have been reviewed for all genetic tests undertaken, including disease-relevant genes in exome sequencing data. The patient is not eligible if they have a molecular diagnosis for their condition.
- Genetic testing should continue according to routine local practice for this phenotype regardless of recruitment to the project; results of these tests must be submitted via the 'Genetic investigations' section of the data capture tool to allow comparison of whole genome sequencing with current standard testing.

Skeletal Muscle Channelopathies prior genetic testing genes

Testing of the following genes should be carried out PRIOR TO RECRUITMENT where this is in line with current local practice:

- Myotonia: *DMPK, CNBP, SCN4A, CLCN1* (including MLPA) - Episodic weakness: *CACNA1S, SCN4A, KCNJ2*

REFERENCES

1. Cagnoli C, Brussino A, Mancini C, Ferrone M, Orsi L, Salmin P, et al. Spinocerebellar Ataxia Tethering PCR: A Rapid Genetic Test for the Diagnosis of Spinocerebellar Ataxia Types 1, 2, 3, 6, and 7 by PCR and Capillary Electrophoresis. J Mol Diagn JMD. 2018 May;20(3):289–97.
2. Nethisinghe S, Lim WN, Ging H, Zeitlberger A, Abeti R, Pemble S, et al. Complexity of the Genetics and Clinical Presentation of Spinocerebellar Ataxia 17. Front Cell Neurosci [Internet]. 2018 [cited 2020 Oct 15];12. Available from: https://www.frontiersin.org/articles/10.3389/fncel.2018.00429/full
3. Warner JP, Barron LH, Brock DJ. A new polymerase chain reaction (PCR) assay for the trinucleotide repeat that is unstable and expanded on Huntington's disease chromosomes. Mol Cell Probes. 1993 Jun;7(3):235–9.
4. Warner JP, Barron LH, Goudie D, Kelly K, Dow D, Fitzpatrick DR, et al. A general method for the detection of large CAG repeat expansions by fluorescent PCR. J Med Genet. 1996 Dec;33(12):1022–6.
5. Campuzano V, Montermini L, Moltò MD, Pianese L, Cossée M, Cavalcanti F, et al. Friedreich's ataxia: autosomal recessive disease caused by an intronic GAA triplet repeat expansion. Science. 1996 Mar 8;271(5254):1423–7.
6. De Bellis A, Quigley CA, Cariello NF, el-Awady MK, Sar M, Lane MV, et al. Single base mutations in the human androgen receptor gene causing complete androgen insensitivity: rapid detection by a modified denaturing gradient gel electrophoresis technique. Mol Endocrinol [Internet]. 1992 Nov 1 [cited 2020 Oct 15];6(11):1909–20. Available from: https://academic.oup.com/mend/article/6/11/1909/2714531
7. Renton AE, Majounie E, Waite A, Simón-Sánchez J, Rollinson S, Gibbs JR, et al. A hexanucleotide repeat expansion in C9ORF72 is the cause of chromosome 9p21-linked ALS-FTD. Neuron [Internet]. 2011 Oct 20 [cited 2020 Oct 15];72(2):257–68. Available from: https://www.ncbi.nlm.nih.gov/pmc/articles/PMC3200438/
8. DeJesus-Hernandez M, Mackenzie IR, Boeve BF, Boxer AL, Baker M, Rutherford NJ, et al. Expanded GGGGCC hexanucleotide repeat in noncoding region of C9ORF72 causes chromosome 9p-linked FTD and ALS. Neuron. 2011 Oct 20;72(2):245–56.
9. Fratta P, Poulter M, Lashley T, Rohrer JD, Polke JM, Beck J, et al. Homozygosity for the C9orf72 GGGGCC repeat expansion in frontotemporal dementia. Acta Neuropathol (Berl) [Internet]. 2013 Sep 1 [cited 2020 Oct 15];126(3):401–9. Available from: https://doi.org/10.1007/s00401-013-1147-0
10. Dolzhenko E, Vugt JJFA van, Shaw RJ, Bekritsky MA, Blitterswijk M van, Narzisi G, et al. Detection of long repeat expansions from PCR-free whole-genome sequence data. Genome Res [Internet]. 2017 Nov [cited 2020 Oct 14];27(11):1895. Available from: https://www.ncbi.nlm.nih.gov/pmc/articles/PMC5668946/
11. Dolzhenko E, Deshpande V, Schlesinger F, Krusche P, Petrovski R, Chen S, et al. ExpansionHunter: a sequence-graph-based tool to analyze variation in short tandem repeat regions. Bioinformatics [Internet]. 2019 Nov 1 [cited 2020 Oct 14];35(22):4754–6. Available from: https://doi.org/10.1093/bioinformatics/btz431
12. Robinson JT, Thorvaldsdóttir H, Wenger AM, Zehir A, Mesirov JP. Variant Review with the Integrative Genomics Viewer. Cancer Res. 2017 01;77(21):e31–4.
13. Rare Disease Eligibility Criteria | Genomics England [Internet]. 2017 [cited 2020 Oct 15]. Available from: https://www.genomicsengland.co.uk/about-genomics-england/the-100000-genomes-project/information-for-gmc-staff/rare-disease-documents/rare-disease-eligibility-criteria/

WGS for neurological diseases group authors:
Ellen M McDonagh PhD[1,2], Antonio Rueda[1], Dimitris Polychronopoulos PhD[1], Georgia Chan PhD[1], Heather Angus-Leppan MBBS (Hons) MSc MD FRACP FRCP[3,4], Kailash P Bhatia MD[5], James E Davison PhD[6], Richard Festenstein FRCP PhD[7,8], Prof Pietro Fratta PhD[9], Paola Giunti MD[5,10], Robin Howard PhD[10], Laxmi Venkata Prasad Korlipara FRCP PhD[11], Matilde Laurá MD PhD[9], Meriel McEntagart MD[12], Lara Menzies PhD[13], Prof Huw R Morris FRCP PhD[5,11], Mary M Reilly MD[9,10], Robert Robinson PhD[14], Elisabeth Rosser FRCP[13], Francesca Faravelli[13], Prof Anette Schrag FRCP PhD[5], Prof Jonathan M Schott FRCP[15], Prof Thomas T Warner FRCP PhD[5,16,17], Prof Nicholas W Wood MD[5,10], David Bourn[18], Kelly Eggleton MSc[19], Robyn Labrum PhD[19], Philip Twiss MSc[20], Stephen Abbs[21], Liana Santos[19], Ghareesa Almheiri MSc[9], Isabella Sheikh MSc[9], Jana Vandrovcova PhD[9], Christine Patch[1], Ana Lisa Taylor Tavares MD[1], Zerin Hyder MD[1], Anna Need PhD[1], Helen Brittain BM BS[1], Emma Baple MBBS PhD[1,22,23], Loukas Moutsianas PhD[1,24], Viraj Deshpande PhD[25], Denise L Perry MS[25], Subramanian S. Ajay PhD[25], Aditi Chawla PhD[25], Vani Rajan MS[25], Kathryn Oprych MD[26,27], Angela Douglas PhD[28], Gill Wilson PhD[29], Prof Sian Ellard PhD[30], Prof I Karen Temple PhD FRCP[31,32], Prof Andrew Mumford PhD FRCP[33], Dominic McMullan[34], Kikkeri Naresh[35], Prof Frances A Flinter MD[36], Jenny C Taylor PhD[37], Lynn Greenhalgh FRCP[28], William Newman PhD[38], Paul Brennan FRCP[39], Prof John A Sayer PhD FRCP[40,41,42], F Lucy Raymond DPhil FRCP FRCPath[43,44], Prof Lyn S Chitty PhD MRCOG[26, 27]

[1]Genomics England, Queen Mary University of London, Charterhouse Square, London, EC1M 6BQ, UK, [2]Open Targets and European Molecular Biology Laboratory - European Bioinformatics Institute (EMBL-EBI), Wellcome Genome Campus, Hinxton, CB10 1SD, UK, [3]Royal Free London NHS Foundation Trust, London, UK, [4]UCL Queen Square Institute of Neurology London, London, UK, [5]Department of Movement and Clinical Neuroscience, UCL Queen Square Institute of Neurology, University College London, London, WC1N 3BG, UK, [6]Metabolic Medicine, Great Ormond Street Hospital for Children NHS Foundation Trust, London, UK, [7]Gene Control Mechanisms and Disease Group, Faculty of Medicine, Department of Brain Sciences and MRC, London, UK, [8]Institute for Medical Sciences, Imperial College London, Hammersmith Hospital, London, UK, [9]Department of Neuromuscular Diseases, UCL Queen Square Institute of Neurology, London, UK, [10]National Hospital for Neurology and Neurosurgery, University College London Hospitals NHS trust, London, UK, [11]UCL Movement Disorders Centre, UCL Queen Square Institute of Neurology, London, WC1N 3BG, UK, [12]St George's, University of London, London, UK, [13]Department of Clinical Genetics, Great Ormond Street Hospital for Children NHS Foundation Trust, London, UK, [14]Department of Paediatric Neurology, Great Ormond Street Hospital for Children National Health Service Trust, London, UK, [15]Dementia Research Centre, UCL Queen Square Institute of Neurology, University College London, London, WCN 3BG, UK, [16]Queen Square Brain Bank for Neurological Disorders, UCL Queen Square Institute of Neurology, London, UK, [17]Reta Lila Weston Institute of Neurological Studies, UCL Queen Square Institute of Neurology, London, UK, [18]Northern Genetics Service, Institute of Genetic Medicine, Central Parkway, Newcastle Upon Tyne, UK, [19]Neurogenetics Unit, National Hospital for Neurology and Neurosurgery, London, UK, [20]East Genomic Laboratory Hub, Addenbrooke's Treatment Centre, Cambridge, UK, [21]East Midlands and East of England NHS Genomic Laboratory Hub, Addenbrooke's Treatment Centre, Cambridge, UK, [22]University of Exeter Medical School, Exeter, EX2 5DW, UK, [23]Peninsula Clinical Genetics Service, Royal Devon & Exeter Hospital (Heavitree), Gladstone Road, Exeter, EX1 2ED, UK, [24]William Harvey Research Institute, Queen Mary University of London, London, EC1M 6BQ, UK, [25]Illumina, Inc, 5200 Illumina Way, San Diego, California, 92122, USA, 5Healx Ltd., Charter House, 66-68 Hills Rd, Cambridge, CB2 1LA, UK, [26]Great Ormond Street Hospital for Children NHS Foundation Trust, London, UK, [27]UCL GOS Institute of Child Health, London, UK, [28]Liverpool Women's NHS Foundation Trust, Liverpool Centre for Genomic Medicine, Crown street, Liverpool, L8 7SS, UK, [29]Sheffield Children's Hospital Clarkson St, Broomhall, Sheffiel, S10 2TH, UK, [30]University of Exeter Medical School, Royal Devon and Exeter Hospital, Wonford, Barrack Road, Exeter, EX2 5DW, UK, [31]Faculty of Medicine,
University of Southampton, University Road, Southampton, SO17 1BJ, UK, [32] Wessex Clinical Genetics Service, University Hospital Southampton NHS Trust, Southampton, UK, [33]School of Cellular and Molecular Medicine, University of Bristol, Bristol, UK, [34]Birmingham Women's Hospital, Mindelsohn Way, Edgbaston, Birmingham, B15 2TG, UK, [35]Imperial College Healthcare NHS Trust Hammersmith Hospital, Du Cane Road, London, W12 0HS, UK, [36]Clinical Genetics Department, Guy's & St Thomas' NHS Foundation Trust, London, SE1 9RT, UK, [37]Oxford NIHR Biomedical Research Centre and Wellcome Centre for Human Genetics, University of Oxford, Oxford, OX3 7BN, UK, [38]Division of Evolution and Genomic Sciences, Faculty of Biology, Medicine and Health, University of Manchester, Manchester, M13 9PL, UK, [39]North East & North Cumbria Genomic Medicine Centre, UK, [40]Translational and Clinical Research Institute, Faculty of Medical Sciences, Newcastle University, Newcastle upon Tyne, NE1 3BZ, UK, [41]National Institute for Health Research Newcastle Biomedical Research Centre, Newcastle upon Tyne, UK, [42]Renal Services, The Newcastle Upon Tyne Hospitals National Health Service Trust, Newcastle

upon Tyne, NE77DN, UK, [43]NIHR BioResource, Cambridge University Hospitals, Cambridge Biomedical Campus, Cambridge, CB2 0QQ, UK, [44]Cambridge Institute for Medical Research, University of Cambridge, Cambridge, CB2 0XY, UK

Genomics England Research Consortium
The members of The Genomics England Research Consortium are:

J. C. Ambrose[1], P. Arumugam[1], E. L. Baple[1], M. Bleda[1], F. Boardman-Pretty[1,2], J. M. Boissiere[1], C. R. Boustred[1], H. Brittain[1], M. J. Caulfield[1,2], G. C. Chan[1], C. E. H. Craig[1], L. C. Daugherty[1], A. de Burca[1], A. Devereau[1], G. Elgar[1,2], R. E. Foulger[1], T. Fowler[1], P. Furió-Tarí[1], J. M. Hackett[1], D. Halai[1], A. Hamblin[1], S. Henderson[1,2], J. E. Holman[1], T. J. P. Hubbard[1], K. Ibáñez[1,2], R. Jackson[1], L. J. Jones[1,2], D. Kasperaviciute[1,2], M. Kayikci[1], L. Lahnstein[1], K. Lawson[1], S. E. A. Leigh[1], I. U. S. Leong[1], F. J. Lopez[1], F. Maleady-Crowe[1], J. Mason[1], E. M. McDonagh[1,2], L. Moutsianas[1,2], M. Mueller[1,2], N. Murugaesu[1], A. C. Need[1,2], C. A. Odhams[1], C. Patch[1,2], D. Perez-Gil[1], D. Polychronopoulos[1], J. Pullinger[1], T. Rahim[1], A. Rendon[1], P. Riesgo-Ferreiro[1], T. Rogers[1], M. Ryten[1], K. Savage[1], K. Sawant[1], R. H. Scott[1], A. Siddiq[1], A. Sieghart[1], D. Smedley[1,2], K. R. Smith[1,2], A. Sosinsky[1,2], W. Spooner[1], H. E. Stevens[1], A. Stuckey[1], R. Sultana[1], E. R. A. Thomas[1,2], S. R. Thompson[1], C. Tregidgo[1], A. Tucci[1,2], E. Walsh[1], S. A. Watters[1], M. J. Welland[1], E. Williams[1], K. Witkowska[1,2], S. M. Wood[1,2], M. Zarowiecki[1]

1. Genomics England, London, UK
2. William Harvey Research Institute, Queen Mary University of London, London, EC1M 6BQ, UK

Declaration of interests for members of the WGS for neurological diseases group
Genomics England is a company wholly owned by the UK Department of Health and Social Care created in 2013 to introduce whole genome sequencing into health care in conjunction with NHS England. All authors affiliated with Genomics England (EMM, AR, DP, GC, CP, ALTT, ZH, AN, HB, EB, and LM) are, or were, salaried by or seconded to Genomics England. VD, DLP, SSA, AC, and VR are employees and shareholders of Illumina. LM reports consultancy from Mendelian Ltd. HM is a co-applicant on a patent application related to C9orf72-Method for diagnosing a neurodegenerative disease (PCT/GB2012/052140). KO is supported by the NIHR Great Ormond Street Hospital Biomedical Research Centre. All other named authors declare no competing interests.

Figure S1. Characteristic pileup graph

A) A characteristic pileup graph illustrating a call in *ATXN2* where the estimated genotype for `GCT` repeat unit is 22/40. Reads supporting each genotype are grouped based on the predicted genotype, in this example in three groups: i) two reads supporting 40 repeat units, in the pathogenic range, on the top of the graph; ii) reads flanking the repeat, supporting > 39 repeat units, in the middle; iii) nine reads supporting 22 repeat units, bottom of the graph. **B)** Schematic representation of the pileup graph. Each read has been coloured according to its sequence content, with blue representing the sequence flanking the repeat, and brown the repeated sequence.

A

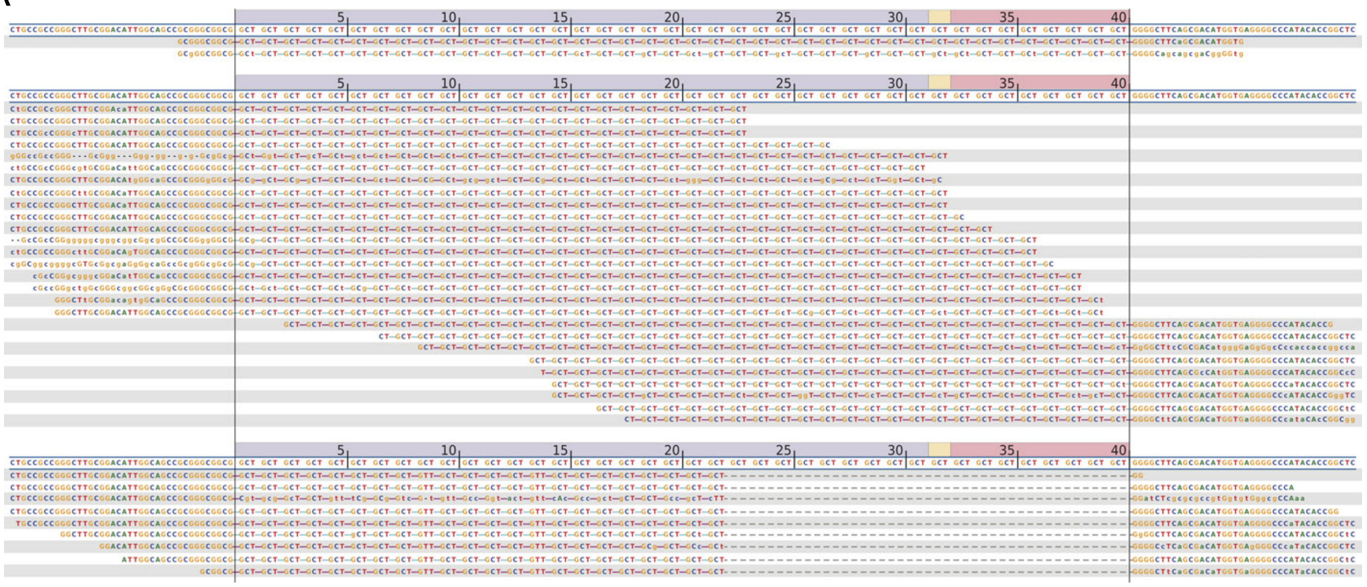

B

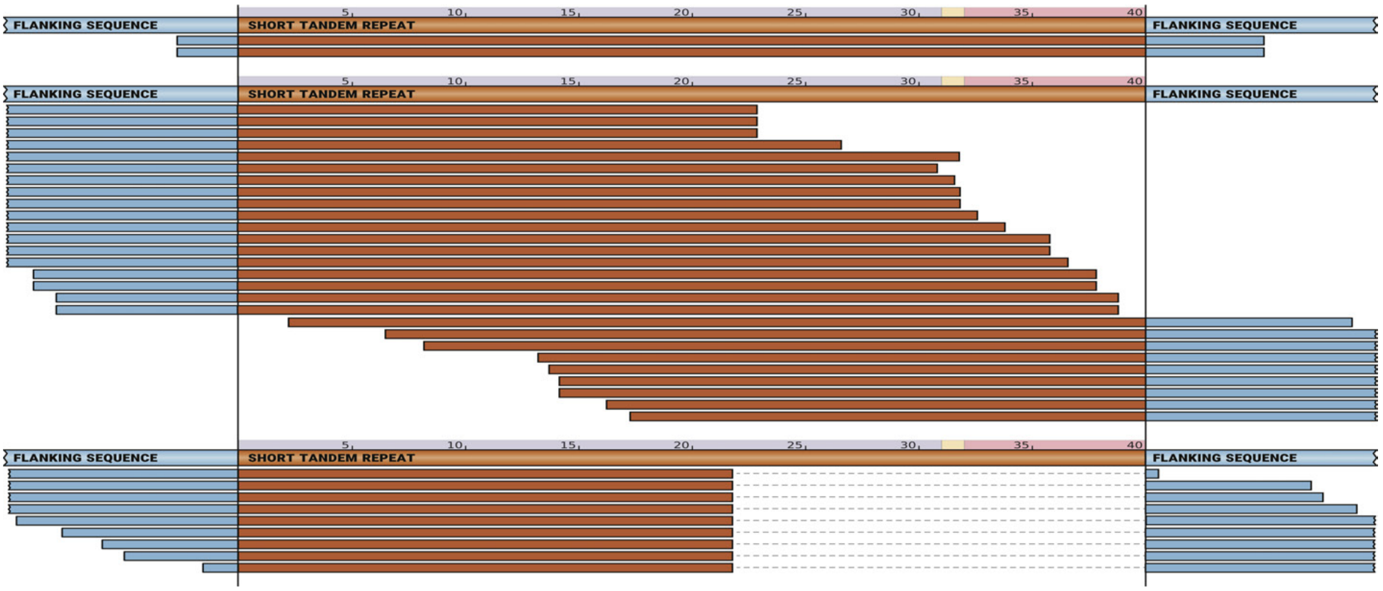

Figure S2. Examples of read pileup graphs

Different scenarios are shown here: **A)** A monoallelic expansion smaller than the read-length. This pileup fully supports the genotype predicted by EH in *ATXN2*. The upper part of the plot shows 2 high-quality reads (nucleotides in uppercase) that completely span the long allele with 40 repeats (green box) and shows sequence flanking the repeats on both sides; the green box below shows 9 reads that completely span the short allele with 22 repeats. The reads in the middle part of the graph contain the repeat and the flanking sequence on one side of the repeat, and hence can be used to estimate the smallest size of the allele. **B)** A monoallelic expansion larger than the read-length. This pileup supports the presence of a large expansion (i.e. larger than the read-length) in a mono-allelic gene, *C9orf72*. 14 reads support the short allele of 2 repeats whilst ~26 reads are fully enriched with `GGCCCC` motif. **C)** A pileup graph of a biallelic expansion which is larger than the read-length. More than 40 reads can be seen fully covered by `GAA`. The presence of some reads with 16 and 19 repeats might represent alleles in mosaicism.

A GE_case_207 - *ATXN2* - Genotype predicted: 22/40 - Genotype predicted after visual QC: 22/40

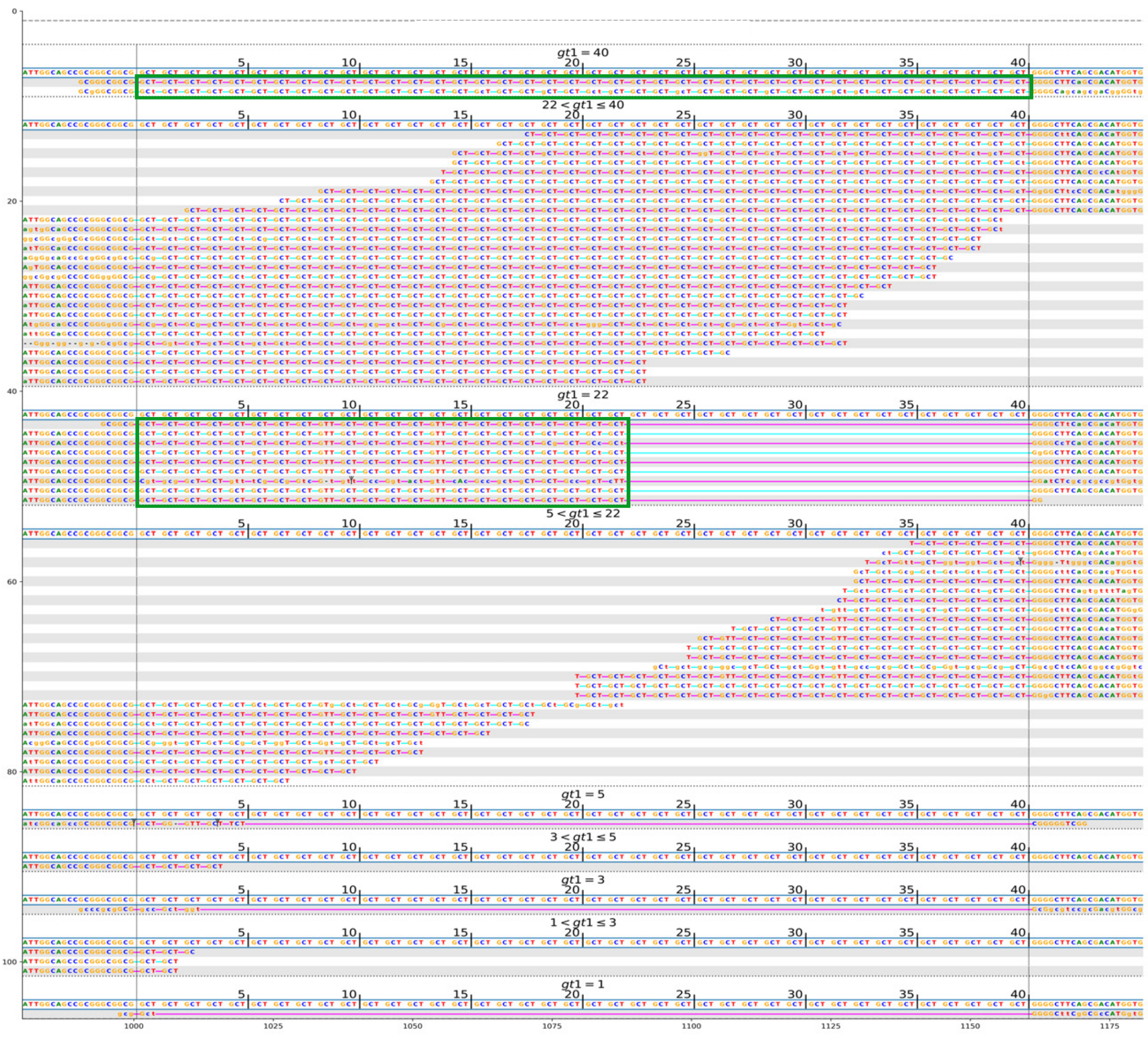

B GE_case_349 - *C9orf72* - Genotype predicted: 5/117 - Genotype predicted after visual QC: 2/EXP

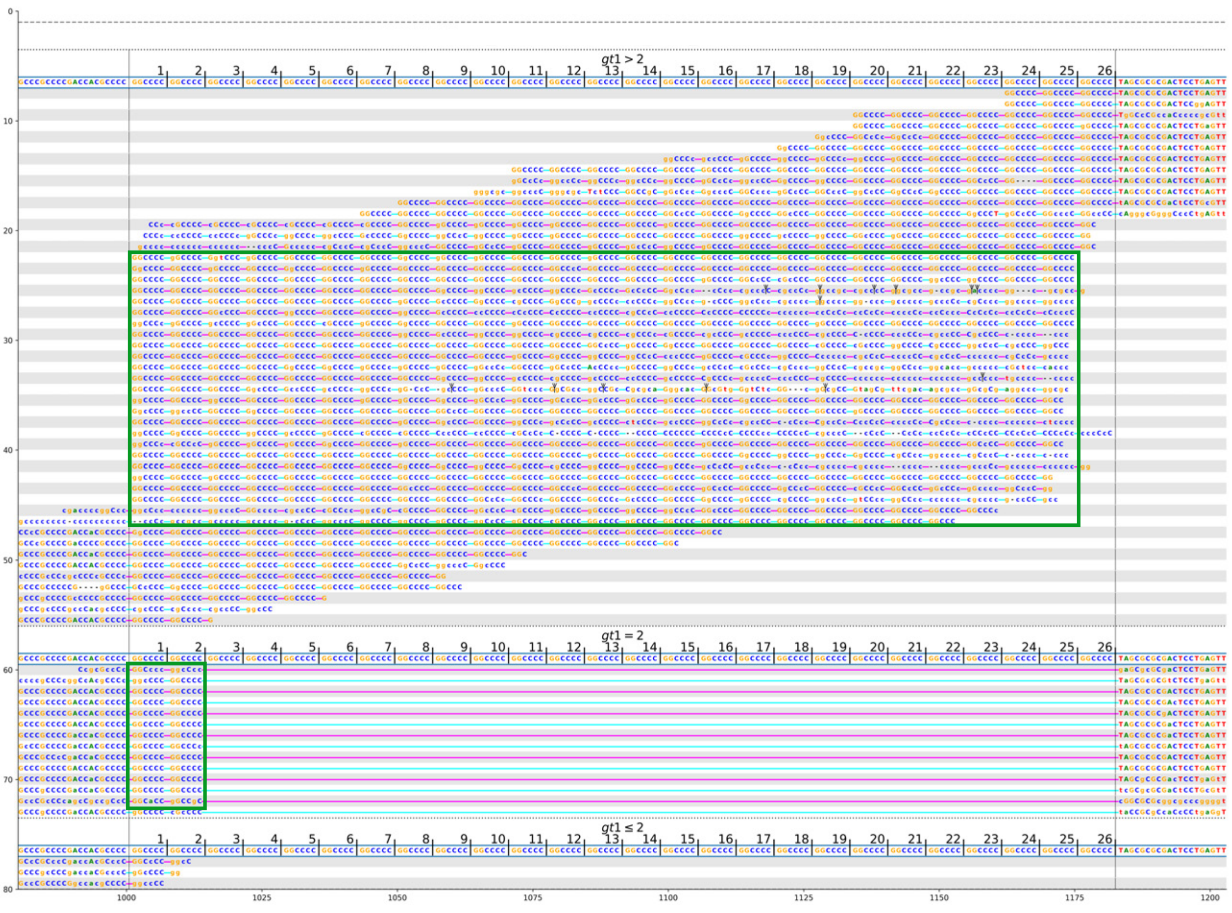

C GE_case_502 - FXN - Genotype predicted: 90/140 - Genotype predicted after visual QC: EXP/EXP

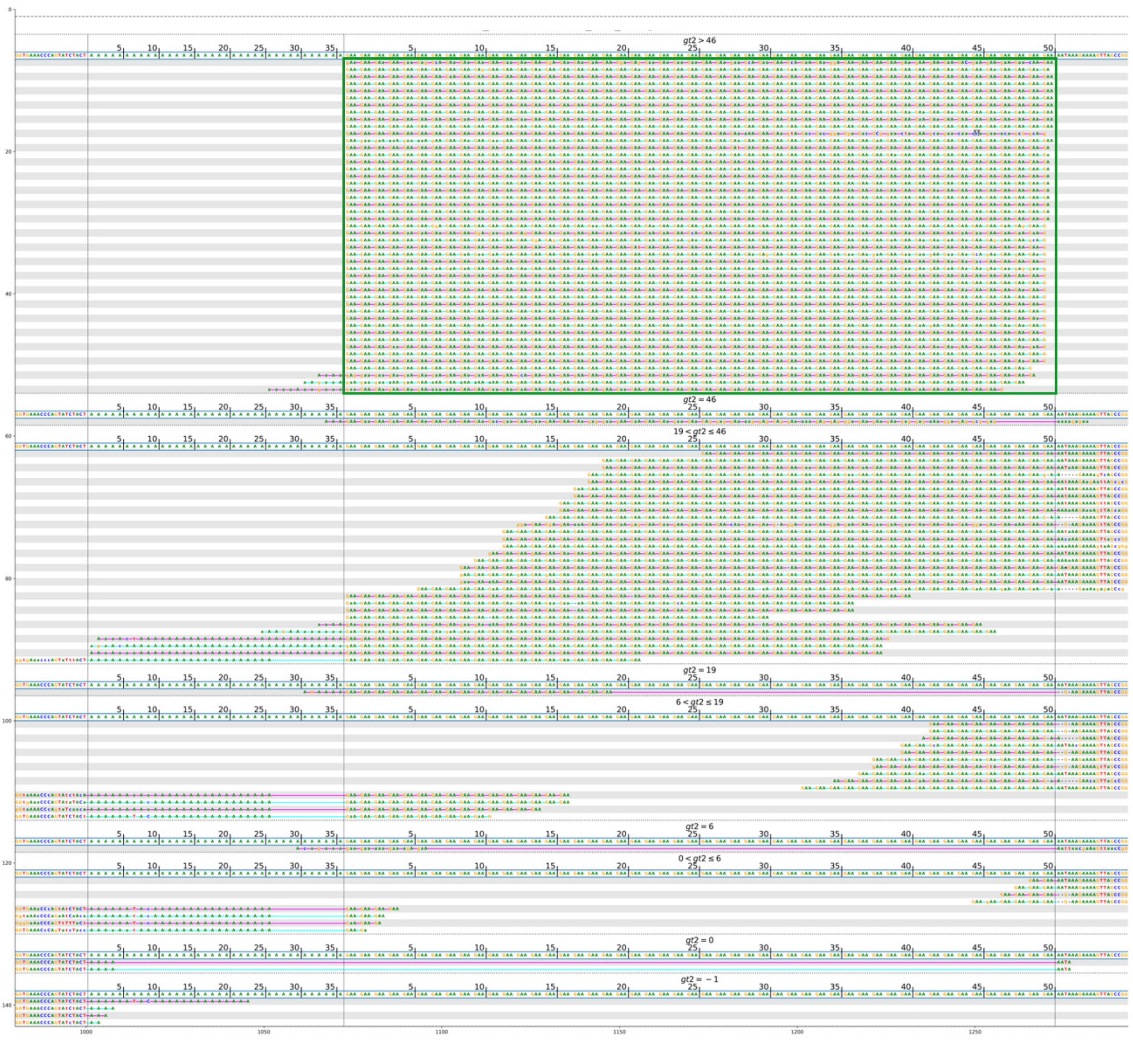

Figure S3. False positive pileups in the RE performance dataset

A total of five false positive alleles from five samples were initially classified by the EHv3.1.2 genotypes, and re-classified after visual inspection of the pileup plot (see **Table S6**). **A)** EH estimations for `GE_case_146` in *ATXN1* are <31,37>. There are three high-quality reads (i.e. upper case) supporting 32 repeats and three reads supporting 31 repeats (highlighted in green rectangle). There is only one read supporting a read with 37 repeats. After visually inspecting this pileup, this case has been classified as normal with <31,32> repeat sizes. PCR sizes were <30,31>. **B)** EH estimations for `GE_case_472` in *FMR1* are <33,57>. The read with 57 `CGG` repeats is fully enriched on low quality bases at the beginning (see the red box outlining the string of lowercase letters) and the call was unreliable (highlighted in red rectangle). PCR sizes for this case were <33,42>. **C)** EH estimations for `GE_case_545` in *HTT* are <18,52>. Originally classified as an expansion but it was determined that the most likely genotype was 18/18 or 18/19 and that the one IRR (red box) was most likely a misaligned read. There are 16 high-quality reads (upper case) supporting 18 repeats. PCR sizes were <18,18>. **D)** EH estimations for `GE_case_559` in *HTT* are <18,36>. Originally classified as 18/36, the most supporting evidence was for alleles of length 18 and 35 and the single read supporting 36 repeats may represent a mosaic. PCR sizes were <18,35>. **E)** EH estimations for `GE_case_96` in *ATXN1* are <36,36>. There are two reads supporting 30 repeats and only one supporting 36 highlighted in green and red rectangles respectively. The bottom part of the pileup shows a large number of reads that partially contain the repeat, and have 2 - 30 repeats maximum. Given that only one read supports 36 repeats, and all the others support 30 or less, this call has been re-classified as normal. PCR sizes were <30,36>. This genome has been sequenced at 125bp read-length.

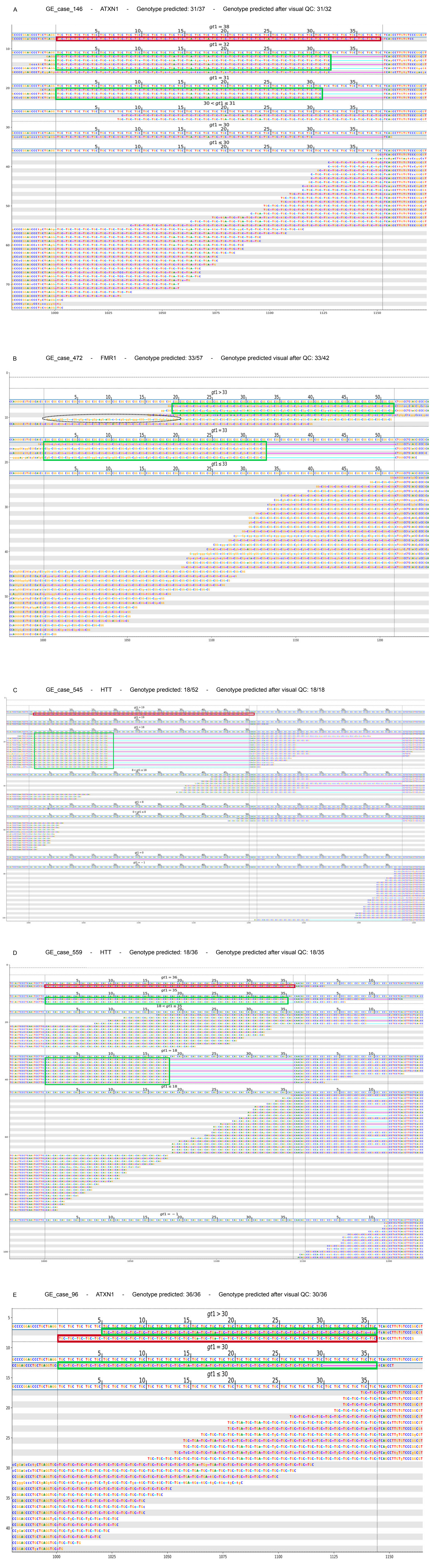

Figure S4. False negative pileups in the RE performance dataset

There are six false negative cases initially detected by EHv3.1.2 in the validation dataset (see **Table S6**). **A-C)** Each of these were originally classified as a monoallelic expansion in *FXN* (highlighted in red rectangles the short allele called by EH). In all three pileup graphs it can be clearly seen that many reads are fully composed of `GAA` repeats (highlighted in green rectangles). There are also one or two reads (highlighted in blue rectangles) that appear to indicate smaller repeats. These are likely due to poor quality at the end of the read where phasing causes the `GAA` sequence to be called `AAA` and so the end of the read appears to be part of the A homopolymer that flanks the repeat. EH calls correctly one expanded allele, and visually inspecting these pileup plots, one can determine both alleles are `expanded`. **D)** EH estimations for `GE_541` in *HTT* are <15,33>. There are many reads supporting the allele with 15 repeats. Upon inspection we decided that the two reads supporting the longer repeat length (36 repeats) were high quality and that this may be a mosaic with 36 repeats for the longest allele. PCR sizes were <15,36>. **E)** EH estimations for `GE_case_101` in *ATXN1* are <32,32>. It can be seen that there are 2 reads supporting 32 repeats and several reads supporting <32 repeats. This sample appears to have low coverage with two reads supporting 33 repeats but more evidence would be needed to make a confident call. This genome has been sequenced at 125bp read-length and PCR exact sizes are not available (<NORMAL, EXP>). This is one of the two FN cases in the final performance dataset after visual inspection (**Table 1**, **Table S6**). **F)** EH estimations for `ICSL_case_144` in *ATXN2* are <22,22> and PCR sizes are <22,42>. This is one of the two FN cases in the final performance dataset after visual inspection (**Table 1**, **Table S6**). The reads in the pileup represent solid 22 repeat-sizes blocks highlighted in green rectangle.

A GE_case_500 - FXN - Genotype predicted: 36/120 - Genotype predicted after visual QC: EXP/EXP

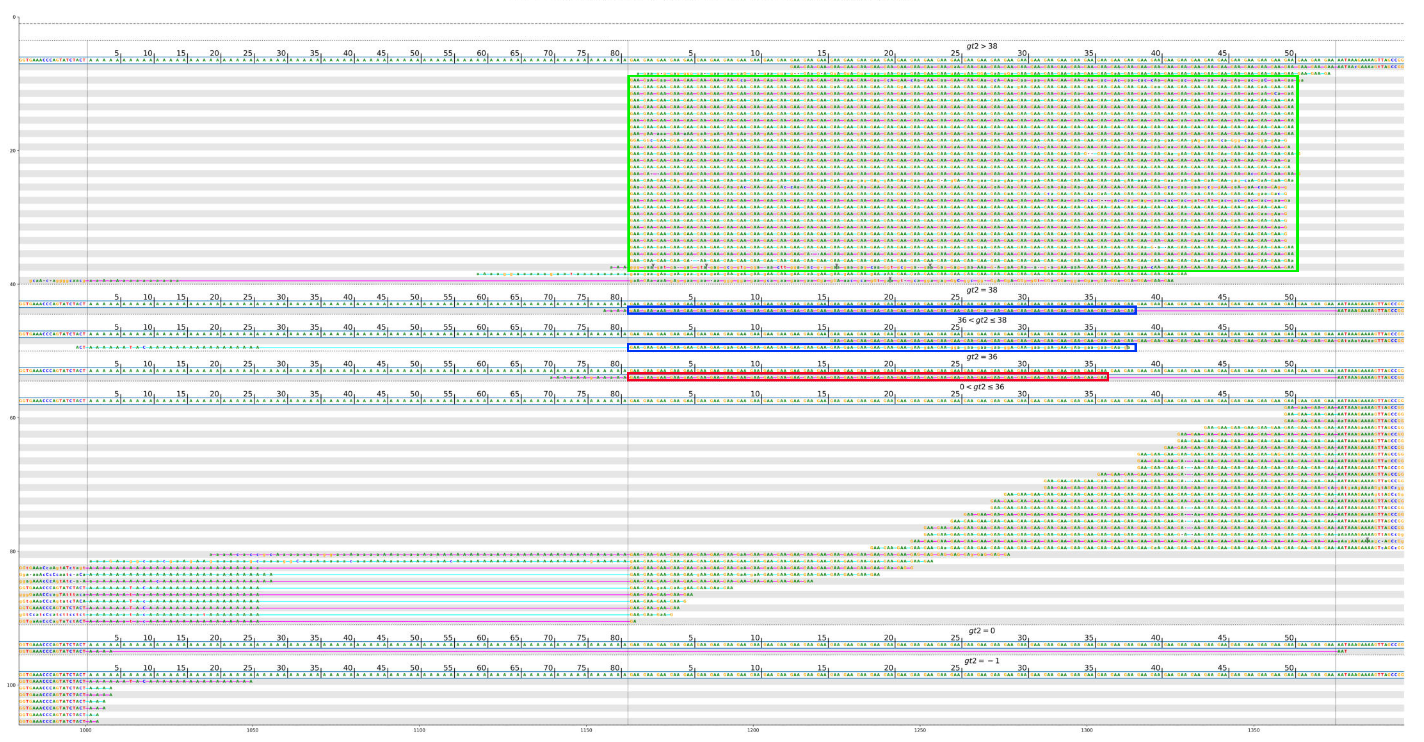

B GE_case_501 - FXN - Genotype predicted: 33/143 - Genotype predicted after visual QC: EXP/EXP

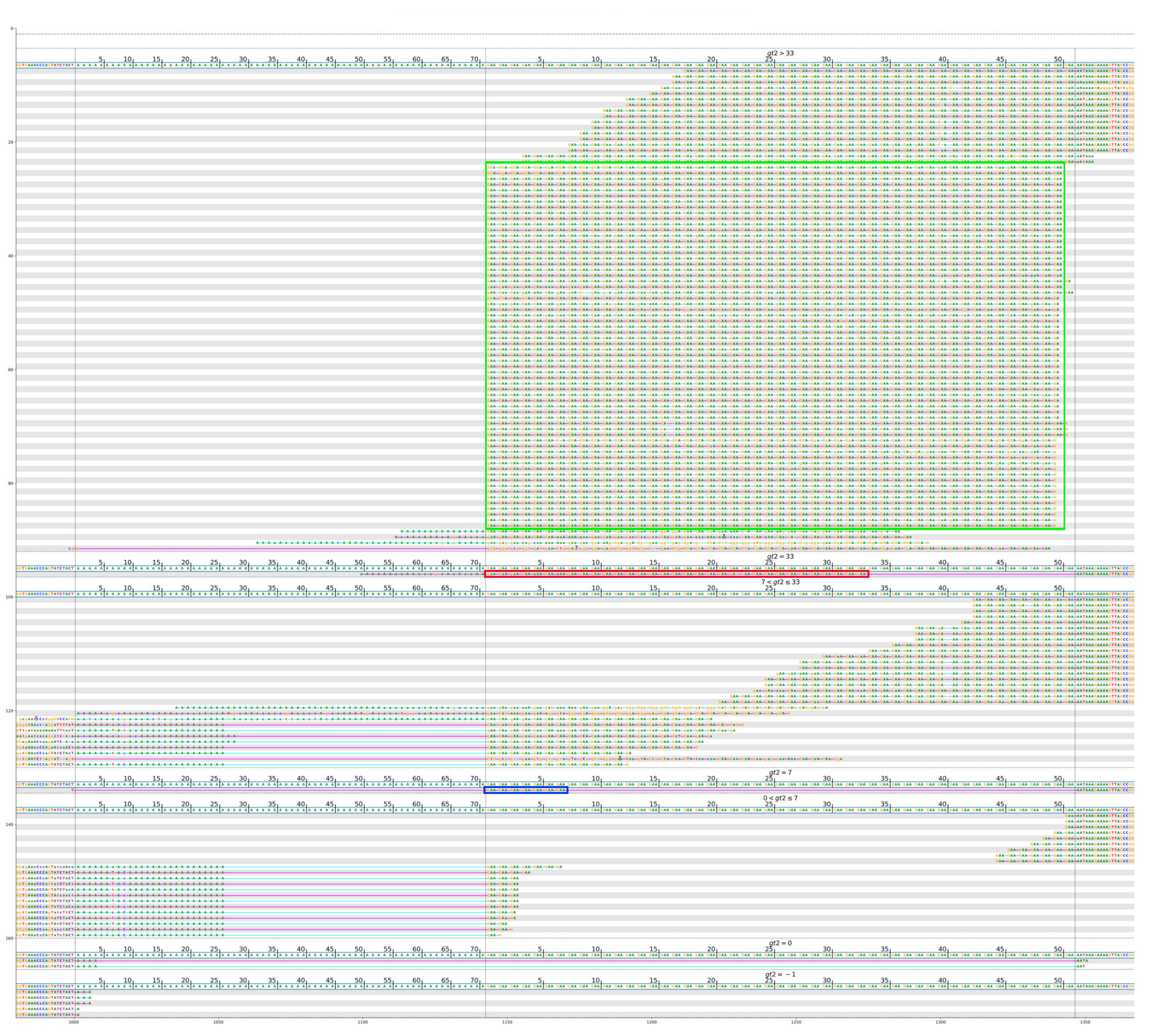

C ICSL_case_72 - FXN - Genotype predicted: 33/113 - Genotype predicted after visual QC: EXP/EXP

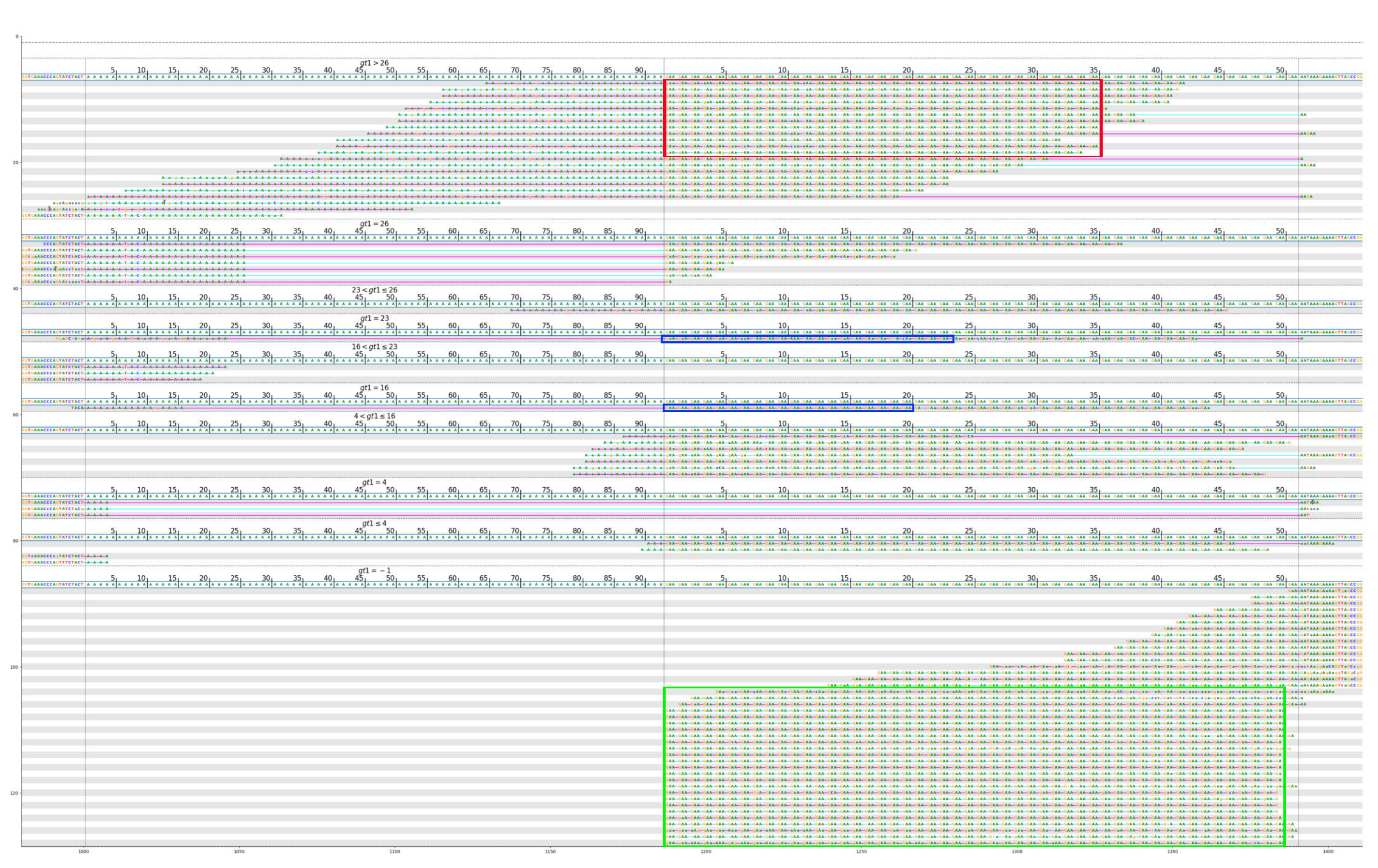

D GE_case_541 - HTT - Genotype predicted: 15/33 - Genotype predicted after visual QC: 15/36

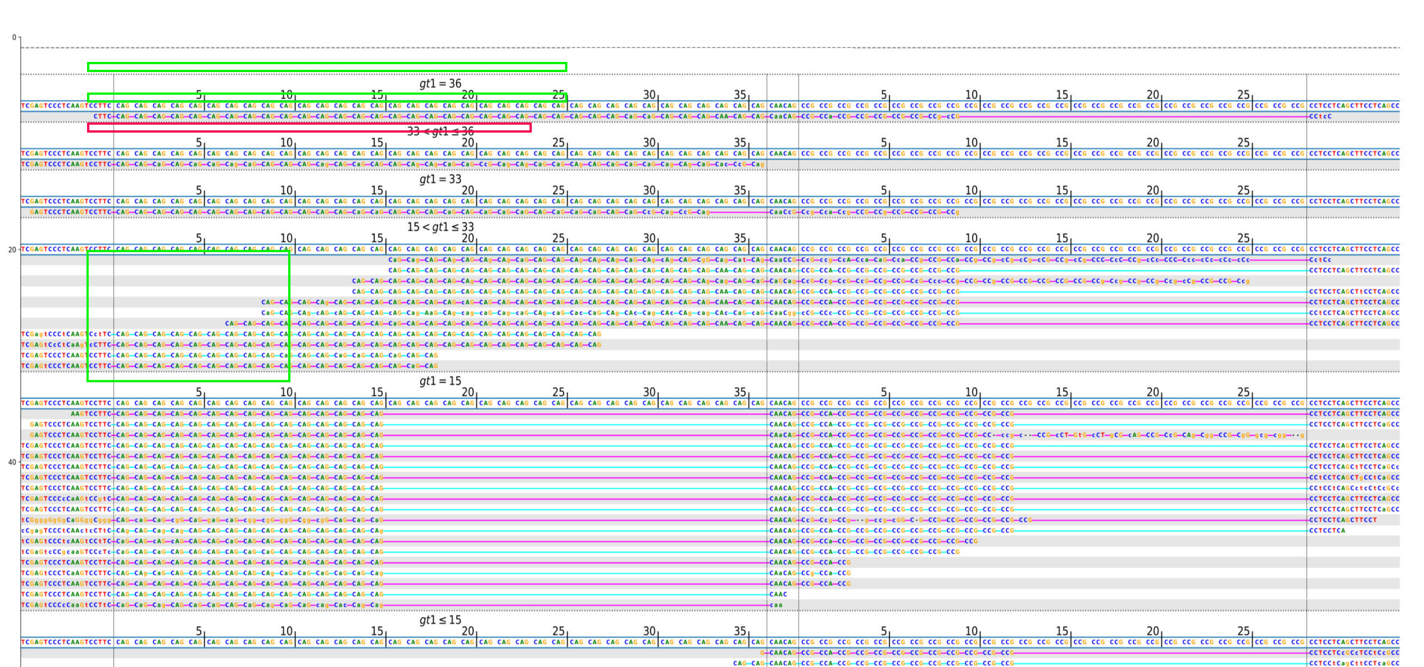

E GE_case_101 - ATXN1 - Genotype predicted: 32/32 - Genotype predicted after visual QC: NA/NA

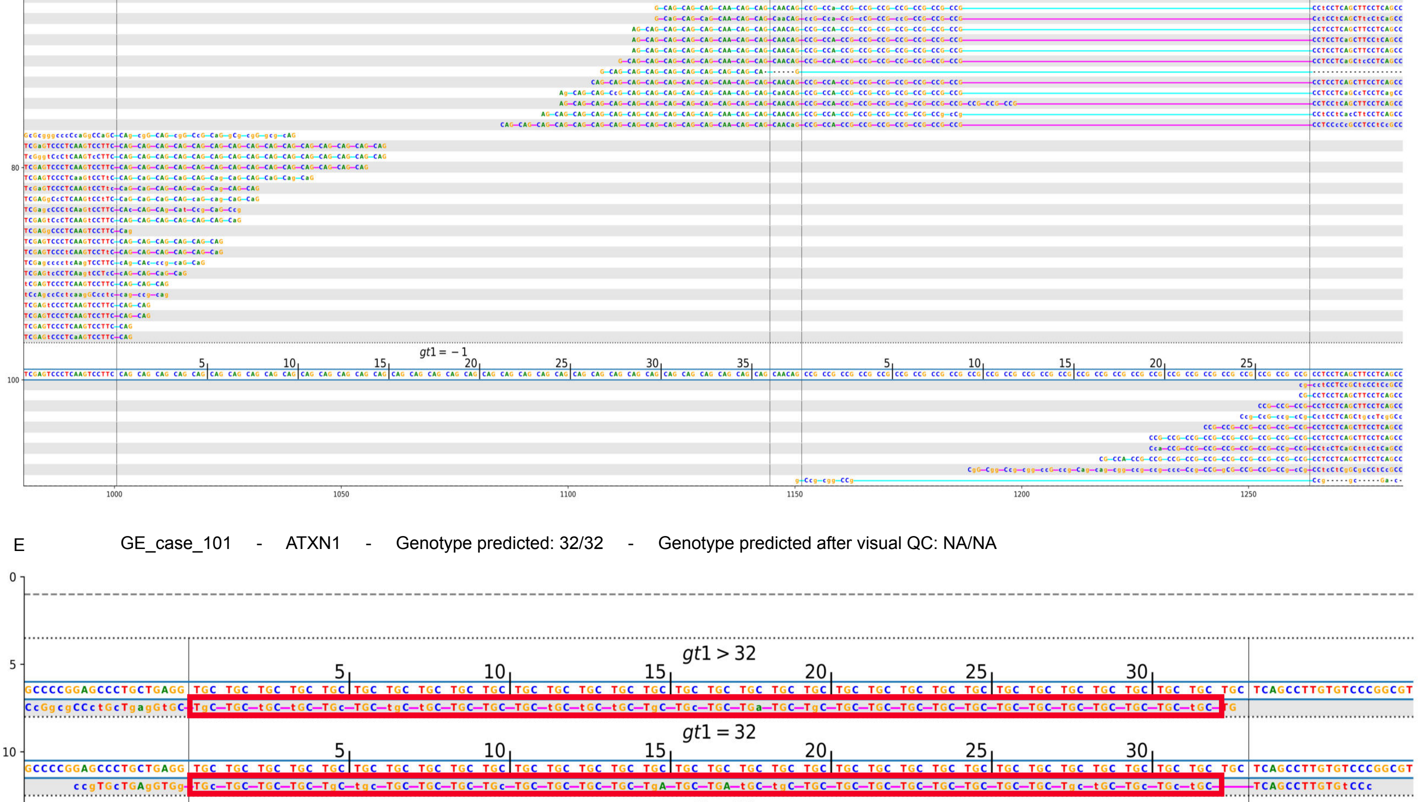

F ICSL_case_144 - ATXN2 - Genotype predicted: 22/22 - Genotype predicted after visual QC: NA/NA

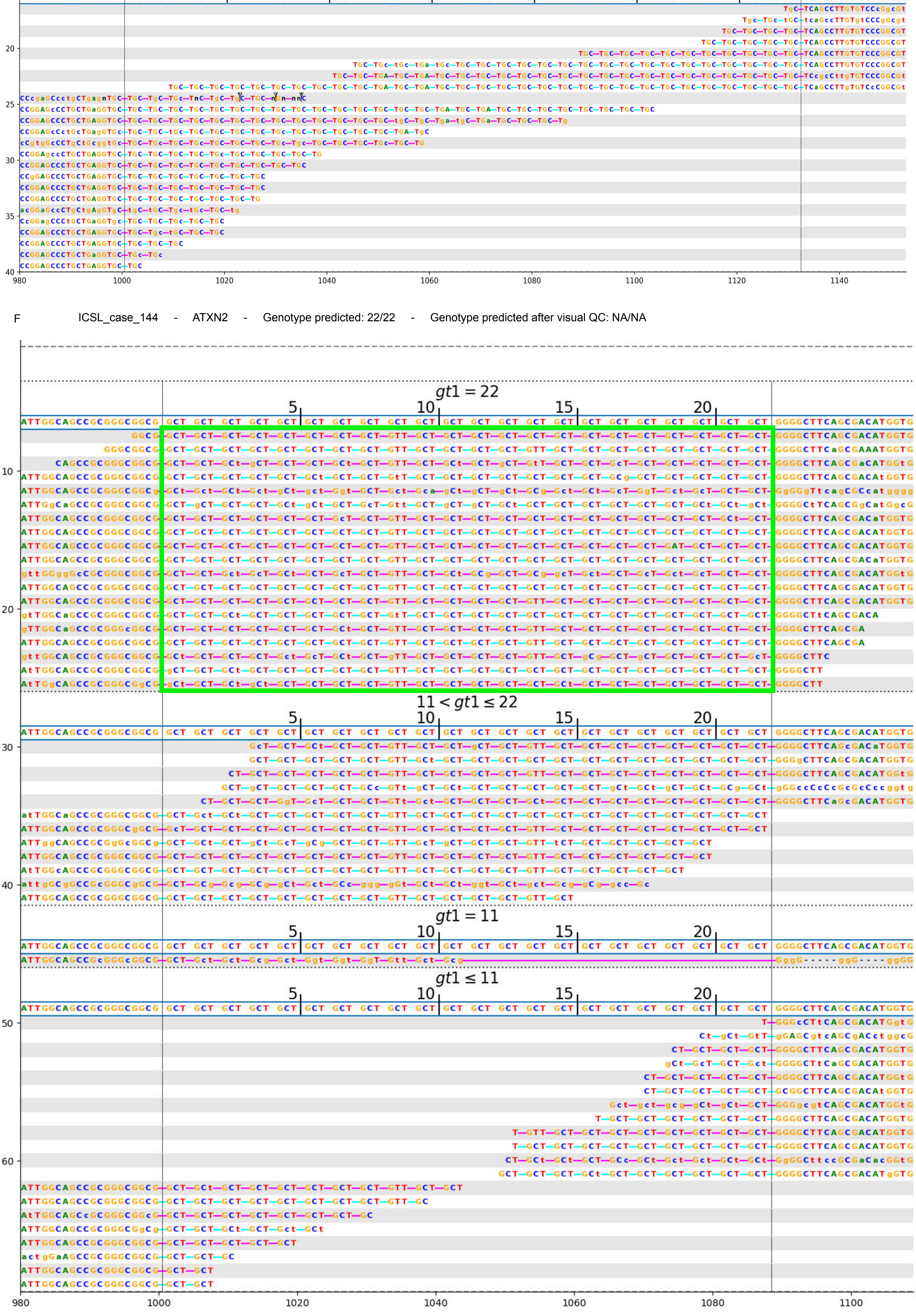

Figure S5. Repeat-size correlation

Bubble plot where PCR and EHv3.1.2 repeat-sizes as base-pair are represented in X and Y axis respectively, and the size of each dot represents the number of cases with the same repeat-size combination. There are two layers, represented by grey and coloured bubbles in background and foreground respectively. The grey bubbles compare the EH sizes before visual inspection against the PCR sizes. The coloured bubbles compare the EH sizes after visual inspection (changing the classification after visual inspection on the five false positives) against the PCR sizes. Dotted vertical red line represents the read-length (i.e. 150bp). **A)** Includes PCR repeat-sizes equal or smaller than read-length, and **B)** Includes all PCR repeat-sizes available against EH estimations. **Figure 2B** contains the breakdown by locus.

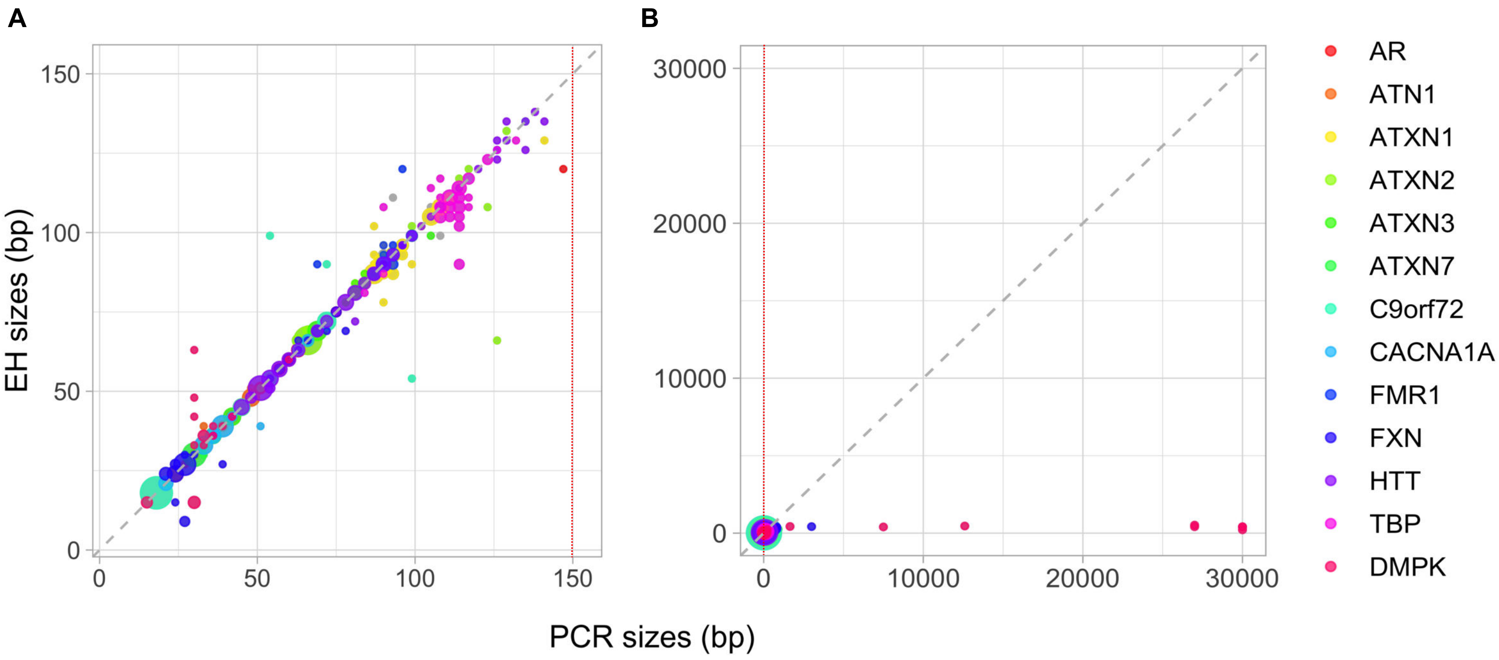

Figure S6. Pileup graphs of not tested genomes

There are a total of 24 genomes that because of lack of DNA have not been validated by PCR. Almost all of them seem to be real expansions and they could potentially been validated. All seem to be potentially expanded alleles. There are four cases that are not very clear: `not_tested_case_17`, `not_tested_case_22`, `not_tested_case_23`, and `not_tested_case_24` have only one read supporting the expanded allele (i.e. borderlines). Absolute numbers are included in **Table S11**.

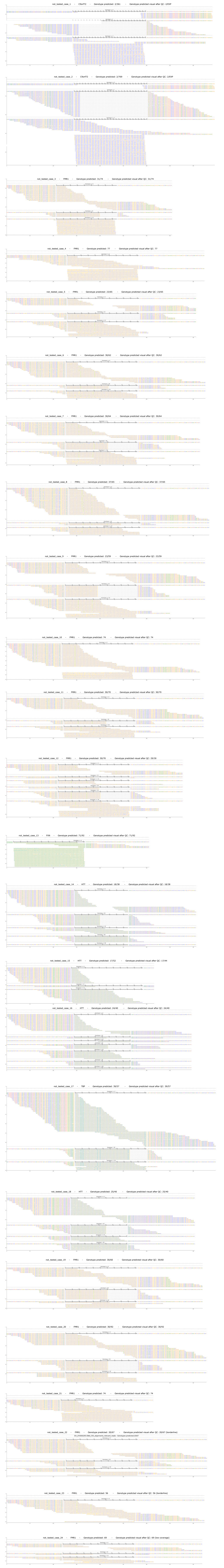

Figure S7. Pileup of false positive calls in the 100,000 Genomes Project

These are the pileup plots for the six cases in the 100,000 Genomes Project initially classified as expanded by EHv2.5.5 that were not orthogonally confirmed. **A)** EH estimations for this case in *ATXN1* are <29,49> and PCR sizes <29,30>. Reads with repeat sizes equal to 29 and 30 can be seen clearly with good flanking regions on both sides of the reads (in green boxes). There are 4 reads (in a blue rectangle) almost fully enriched with `CTG` with 48 repeats. This could be a misalignment. **B)** EH and PCR repeat-sizes for this case in *ATXN2* are <22,48> and <22,22> respectively. There are 28 reads supporting 22 repeat-sizes, while 3 reads support a larger allele with 48 repeats. This could be a misalignment. We here note that the re-analysis of these samples with EHv3.1.2 shows repeat sizes in the normal range, being of <31,31> for *ATXN1* in case A and <22,22> for *ATXN2* in case B. **C)** EH estimation for this case in *FMR1* is 61 whilst PCR size is 51. There are 6 reads (in green box) with good quality `CGG` repeats, but the end of the read is fully covered by low quality bases (i.e. in lowercase). **D)** EH estimations for this case in *FMR1* are <30,62> and PCR sizes <30,53>. There are three reads supporting 30 repeats (green box), and only one read (red box) fully covered with `CGG`. There is not enough read coverage in order to make a good call. **E)** EH estimation for this case in *FMR1* is 68 while PCR is 48. Similar to case E, there is not enough read-coverage in order to make a call. **F)** EH estimation for this case in *FMR1* is 60 whilst PCR is 54. The four reads fully covered by `CGG` (in red box) have low quality bases at the beginning of the read, showing that expansion might be smaller than what EH is estimating.

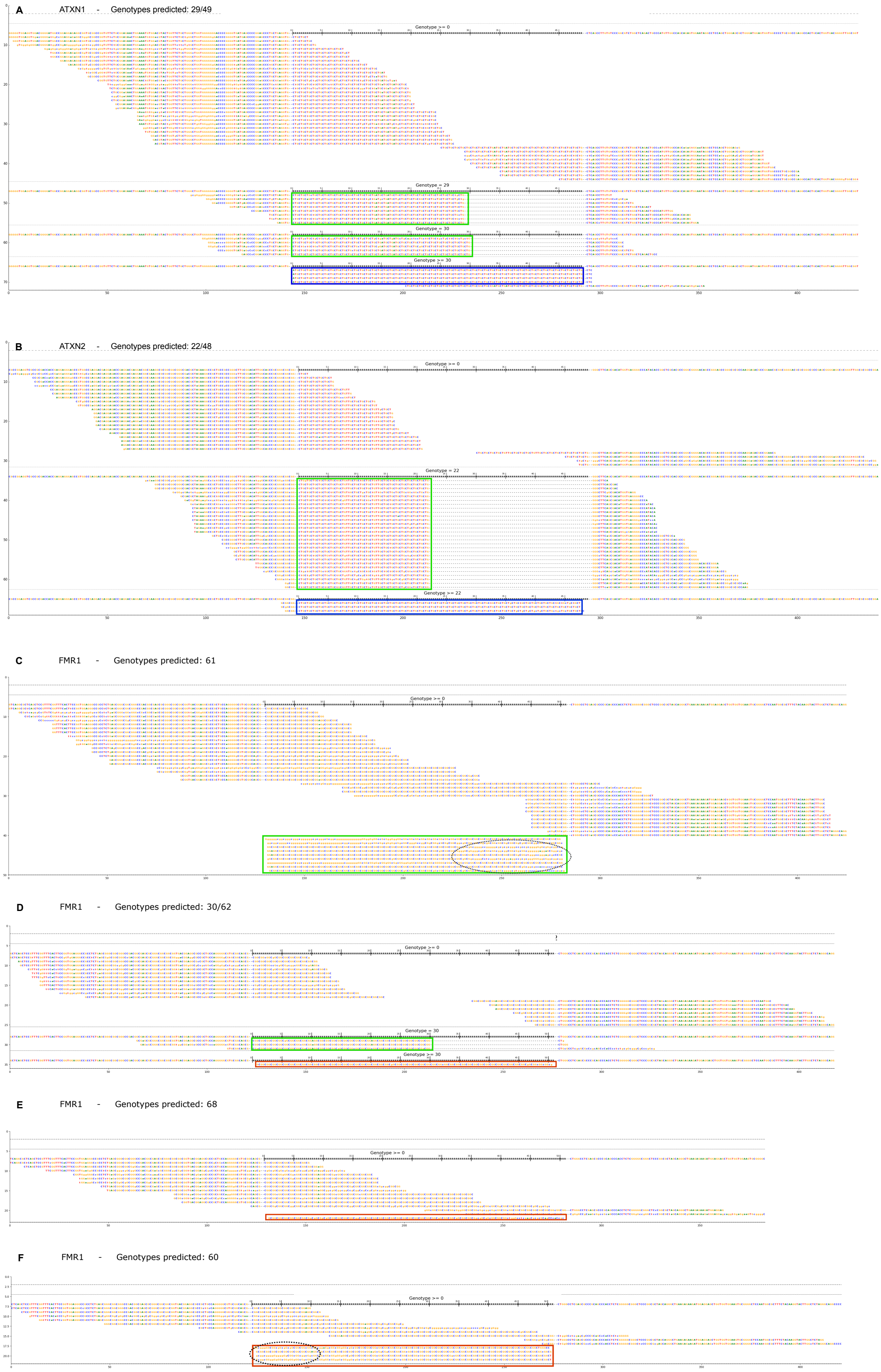

Figure S8. Overview of confirmed repeat expansions in the neurodegenerative panel cohort (Panel A).
Total number of patients tested (grey bars), and patients with repeat expansions confirmed by PCR (coloured bars), per clinical presentation. E.O. = early onset. Patients were recruited to the 100,000 Genomes Project after standard of care genetic testing; therefore, the proportion of repeat expansions identified do not reflect diagnostic yield in an unselected cohort of patients, but rather an increase in the diagnostic yield compared to standard of care NHS testing. Please note different scales to the left and to the right of 0 on the x axis.

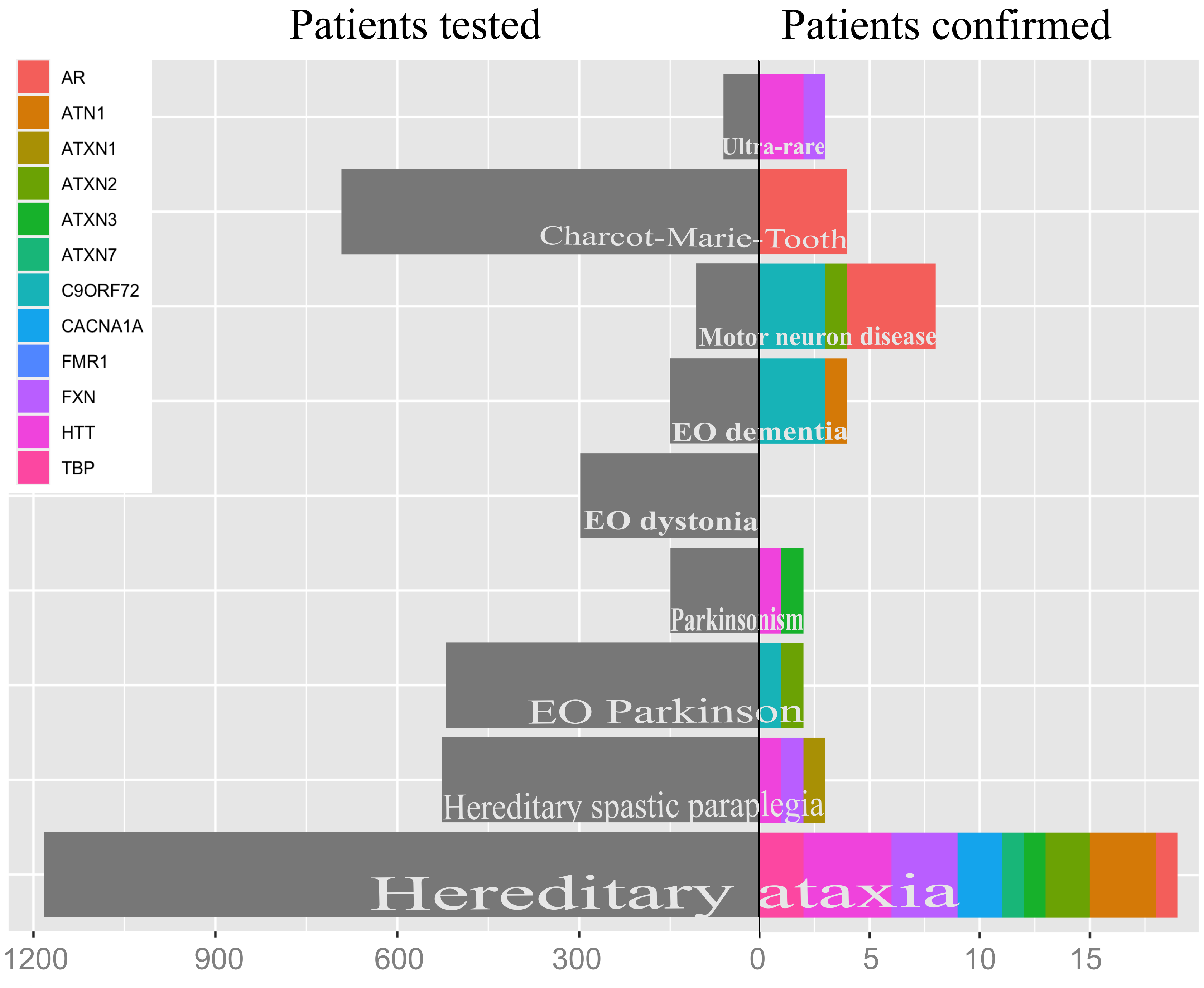

Figure S9. Suggested clinical-diagnostic workflow

This diagram presents a generalised clinical workflow for the detection and reporting of short tandem repeats from whole genome sequencing data. In brief, peripheral blood is collected in an EDTA tube, DNA is prepared using a PCR-free library preparation method and sequencing is performed to a minimum of ~30x depth. We note that STR calling is improved with both greater read depth and longer sequencing reads (e.g. paired-end 150bp reads). Read alignment is performed using a well-established mapping algorithm or genomic analysis package, including BWA and DRAGEN. Both the mapped and unmapped reads, i.e. those that do not align to the reference genome, are then utilised by the ExpansionHunter software package to interrogate for expanded alleles at a minimum of 13 loci. If Expansion Hunter does not identify any expansion, but there is still clinical or laboratory suspicion of a RE disorder, visual inspection of the validated STR loci is recommended. Visual inspection is also recommended for any locus that EH detects as potentially expanded. If the quality of the reads and the associated EH call is of high quality the sample should be sent for orthogonal characterisation. If the reads and the associated EH call is of poor quality, it is recommended that only those patients with strong phenotypic overlap are sent for orthogonal characterisation.

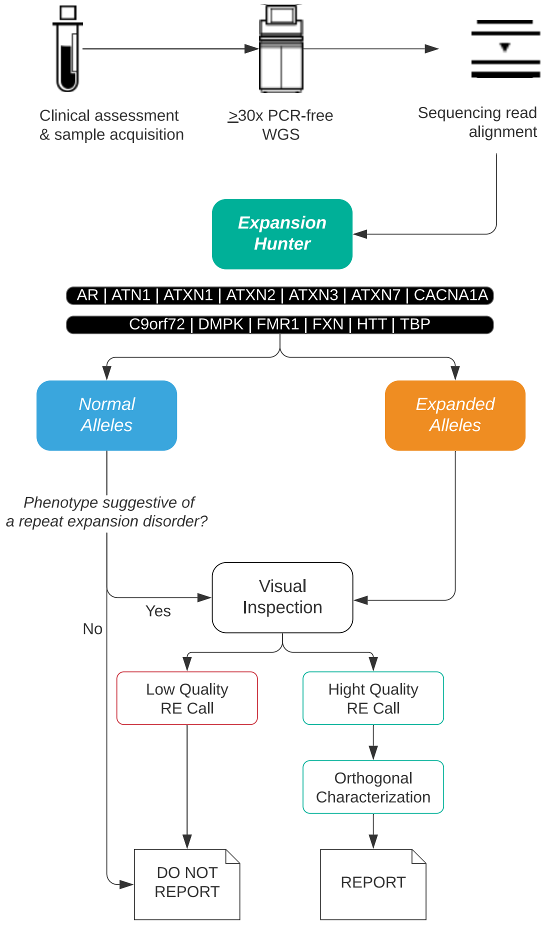

Table S1. Repeat expansion diagnostic accuracy dataset: total number of tests per repeat expansion gene. Count of tests carrying alleles in the non-expanded, intermediate and full-mutation range, sequenced by Genomics England (GE) and Illumina Clinical Services Laboratory (ICSL). Note some tests might have only one allele, particularly, male samples in genes located in X chromosome (i.e. *AR*, and *FMR1*). Thus, each number counts a genome reported as having an allele with a repeat size within the non-expanded/intermediate/full-mutation threshold defined in Table S5.

Disease	STR gene	Number of tests (Non-expanded; Intermediate; Full-mutation)		Number of alleles (Non-expanded; Intermediate; Full-mutation)	
		**GE** (n=634)	**ICSL** (n=159)	**GE** (n=1233)	**ICSL** (n=309)
**TOTAL**		**577; 23; 34**	**33; 1; 125**	**1172; 25; 36**	**149; 17; 143**
Spinal and bulbar muscular atrophy of Kennedy	*AR*	25; 0; 5	1; 0; 2	34; 0; 5	2; 0 ;2
Dentatorubral-pallidoluysian atrophy	*ATN1*	44; 0; 3	-	91; 0; 3	-
Spinocerebellar ataxia 1	*ATXN1*	63; 14; 1	4; 0; 1	141; 14; 1	9; 0; 1
Spinocerebellar ataxia 2	*ATXN2*	55; 1; 5	3; 0; 3	116; 1; 5	9; 0; 3
Spinocerebellar ataxia 3	*ATXN3*	52; 0; 3	0; 0; 2	107; 0; 3	2; 0; 2
Spinocerebellar ataxia 7	*ATXN7*	52; 0; 1	-	105; 0; 1	-
Frontotemporal dementia and/or amyotrophic lateral sclerosis 1	*C9orf72*	62; 1; 5	-	130; 1; 5	-
Spinocerebellar ataxia 6	*CACNA1A*	53; 1; 1	3; 0; 8	108; 1;1	14; 0; 8
Myotonic dystrophy 1	*DMPK*	-	0; 0; 42	-	42; 0; 42
Fragile X syndrome	*FMR1*	23; 2; 2	0; 0; 16	36; 2; 2	9; 0; 16
Friedreich ataxia	*FXN*	24; 0; 4	22; 0; 12	48; 0; 8	22; 0; 46
Huntington disease	*HTT*	67; 2; 4	0; 1; 39	140; 2; 4	40; 1; 39
Spinocerebellar ataxia 17	*TBP*	57; 2; 0	-	116; 2; 0	-

Table S2. Primers used for each PCR assay by the Neurogenetics Laboratory at the National Hospital for Neurology and Neurosurgery.

***ATXN1* Tethered RP-PCR**	
NED-TTTGCTGGAGGCCTATTCCAC TCT	GAGCCCTGCTGAGGTGCTGCTGCTGCTGCTG
***ATXN2* Tethered RP-PCR**	
VIC-TTTCGGCGGCTCCTTGGTCTC	AGCCGCGGGCGGCGGCTGCTGCTGCTGCTG
***ATXN3* Tethered RP-PCR**	
6FAM-AGTCCAGTGACTACTTTGAT TCG-	GTCCTGATAGGTCCCCCTGCTGCTGCTGCTG’
***CACNA1A* Tethered RP-PCR**	
VIC-TTTTTCCCCTGTGATCCGTAA GG	CGGCCTGGCCACCGCCTGCTGCTGCTGCTG
***ATXN7* Tethered RP-PCR**	
6FAM-TTTGAAAGAATGTCGGAGC GGG	CTGCGGAGGCGGCGGCTGCTGCTGCTGCTG
**ATXN7 Flanking PCR**	
6FAM-CACGACTCTCCCAGCATCACTT	TGTTACATTGTAGGAGCGGAA
TBP	
FAM GATGCCTTATGGCACTGGACTG	CTGCTGGGACGTTGACTGCTG
***HTT* PCR1**	
6FAM-CCTTCGAGTCCCTCAAGTC CTT	GGCGGTGGCGGCTGTTGCTGCTGCTGCTGC
***HTT* PCR2**	
6FAM-5'-CCTTCGAGTCCCTCAAGT CCTT	CGGCTGAGGCAGCAGCGGCTGT
***FXN* Flanking PCR**	
6FAM-GGGATTGGTTGCCAGTGCT TAAAAGTTAG	GATCTAAGGACCATCATGGCCACACTTGCC
***FXN* RP-PCR**	
GCTGGGATTACAGGCGCGCGA	Repeat-binding: TACGCATCCCAGTTTGAGACGGAAGAAGAA GAAGAAGAAGAA<br>Non-genomic: 6FAM-TACGCATCCCAGTTTGAGACG
AR	
6FAM-GCCTGTTGAACTCTTCTGA GC	GCTGTGAAGGTTGCTGTTCCTC
ATN1	
6FAM CACCAGTCTCAACACATCACCATC	CCTCCAGTGGGTGGGGAAATGCTC
***C9orf72* RP-PCR1**	
6FAM-AGTCGCTAGAGGCGAAAGC	Repeat-binding: TACGCATCCCAGTTTGAGACGGGGGCCGGGGC CGGGGCC

	Non-genomic:<br>TACGCATCCCAGTTTGAGACG
***C9orf72* RP-PCR 2**	
6FAM-CAAGGAGGGAAACAACCG<br>CAGCC	Repeat-binding:<br>CAGGAAACAGCTATGACCGGGCCCGCCCCGAC<br>CACGCCCCGGCCCCGGCCCCGG<br>Non-genomic: CAGGAAACAGCTATGACC
***C9orf72* Flanking PCR**	
6FAM-CAAGGAGGGAAACAACCG<br>CAGCC	GCAGGCACCGCAACCGCAG

Table S3. **Summary of the sequence data used in this study**. Whole genome sequencing data was generated in the Illumina Clinical Services Laboratory (ICSL; San Diego, CA USA) or in the Illumina Laboratory Services high-throughput genome facility (Hinxton, South Cambridgeshire UK) for Genomics England (GE).

**Laboratory**		ICSL	GE	GE	GE
**Chemistry**		Truseq PCR-free	Truseq PCR-free	Truseq PCR-free	Truseq PCR-free
**Read length**		2x150bp	2x150bp	2x150bp	2x125bp
**Genome build**		37.1	38	37.1	37.1
**Diagnostic accuracy dataset**	ExpansionHunter version	3.1.2	3.1.2	3.1.2	3.1.2
	Number of patients	150	165	53	36
**100,000 Genomes Project undiagnosed patients**	ExpansionHunter version	-	2.5.5	2.5.5	2.5.5
	Number of patients	-	9598	1746	287

Table S4. Repeat expansion diagnostic accuracy dataset including GE and ICSL Panels. Each row corresponds to a case (`validation_id`) which has been tagged as a unique patient ID associated with a genome and a locus. For each case the genome's read_length, gender, PCR sizes for each allele (numeric value if there is such information or `exp` and `non-exp` for expansions and non-expanded alleles respectively), ExpansionHunter version 2.5.5 ('EHv255') and ExpansionHunter version 3.1.2 ('EHv312') average repeat-sizes estimations for each allele, as well as the classification for both ExpansionHunter versions before and after visual inspection when comparing to PCR sizes can be found. `Locus coverage` shows the read coverage on each gene for a particular genome. The boolean value (Yes or No) in `repeat_sizing_test_any` represents whether the genome is included (`Yes`) or not (`No`) in the repeat sizing analysis. The concordance test is calculated for each allele, `included_in_concordance_test_PCR_smaller_read-length_aX` columns are `Yes` whether the allele is included in the PCR-EH repeat-size concordance calculation. Columns called `concordance_PCR_length_EHv312_length_1_repeat_error_aX` represent the difference with 1 repeat error between PCR and EH sizes (see Table S7).

Due to formatting and display issues, as well as facilitating a more detailed examination, this table is available at the following web address: https://drive.google.com/file/d/1fwvITwwLk-EZiqdkuGds-Z69xyid4fqf/view?usp=sharing

Table S5. **Repeat-size thresholds for premutation and full-mutation.** Thresholds defined for non-expanded and full-mutation repeat-sizes (number of nucleotides) for each repeat expansion disease. Intermediate numbers are within these both cut-off values.

**Disease**	**Prevalence**	**Locus**	**Premutation repeats cut-off (# nucleotides)**	**Full Mutation repeats cut-off (# nucleotides)**
Dentatorubral-pallidoluysian atrophy	<1:100,000	*ATN1*	>34 (102)	>=48 (144)
Fragile X syndrome	1:7,000 males<br>1:11,000 females	*FMR1*	>55 (165)	>=200 (600)
Friedreich ataxia	2-4:100,000	*FXN*	>44 (132)	>=66 (198)
C9orf72-related FTD or ALS	0.4-1.5:100,000	*C9orf72*	>30 (180)	>=60 (360)
Huntington disease	9.71: 100,000	*HTT*	>35 (105)	>=40 (120)
Myotonic dystrophy 1	1:20,000	*DMPK*		>=50 (150)
Spinocerebellar ataxia 1	1-2:100,000	*ATXN1*	>35 (105)	>=44 (132)
Spinocerebellar ataxia 2	1-2:100,000	*ATXN2*	>31 (93)	>=33 (99)
Spinocerebellar ataxia 3	<1:100,000	*ATXN3*	>43 (129)	>=60 (180
Spinocerebellar ataxia 6	<1:100,000	*CACNA1A*	>17 (51)	>=20 (60)
Spinocerebellar ataxia 7	<1:100,000	*ATXN7*	>34 (102)	>=36 (108)
Spinocerebellar ataxia 17	<1:100,000	*TBP*	>41 (123)	>=49 (147)
Spinal and bulbar muscular atrophy of Kennedy	2:100,000 males	*AR*	>34 (102)	>=38 (114)

Table S6. Performance of ExpansionHunter in the repeat expansion diagnostic accuracy dataset. Estimates have been generated with ExpansionHunter version 3.1.2. TN= true negative; FP = false positive; TP = true positive; FN = false negative. Two tables are shown below, computing performance by allele and by patient or sample. See Table S16 for performance by ExpansionHunter version 2.5.5

Per allele:	**EHv3.1.2**						**EHv3.1.2 after visual QC**					
	**TN**	**FP**	**TP**	**FN**	**Sensitivity**	**Specificity**	**TN**	**FP**	**TP**	**FN**	**Sensitivity**	**Specificity**
AR	36	0	7	0	100%	100%	36	0	7	0	100%	100%
ATN1	91	0	3	0	100%	100%	91	0	3	0	100%	100%
ATXN1	148	2	15	1	93·8%	98·7%	150	0	15	1	93·8%	100%
ATXN2	125	0	8	1	88·9%	100%	125	0	8	1	88·9%	100%
ATXN3	109	0	5	0	100%	100%	109	0	5	0	100%	100%
ATXN7	105	0	1	0	100%	100%	105	0	1	0	100%	100%
C9orf72	130	0	6	0	100%	100%	130	0	6	0	100%	100%
CACNA1A	122	0	10	0	100%	100%	122	0	10	0	100%	100%
DMPK	42	0	42	0	100%	100%	42	0	42	0	100%	100%
FMR1	44	1	20	0	100%	97·8%	45	0	20	0	100%	100%
FXN	70	0	51	3	94·4%	100%	70	0	54	0	100%	100%
HTT	178	2	45	1	97·8%	98·9%	180	0	46	0	100%	100%
TBP	116	0	2	0	100%	100%	116	0	2	0	100%	100%
**TOTAL**	**1316**	**5**	**215**	**6**	**97·3%**	**99·6%**	**1321**	**0**	**219**	**2**	**99·1%**	**100%**

Per patient:	**EHv3.1.2**						**EHv3.1.2 after visual QC**					
	**TN**	**FP**	**TP**	**FN**	**Sensitivity**	**Specificity**	**TN**	**FP**	**TP**	**FN**	**Sensitivity**	**Specificity**
AR	26	0	7	0	100%	100%	26	0	7	0	100%	100%
ATN1	44	0	3	0	100%	100%	44	0	3	0	100%	100%
ATXN1	66	1	15	1	93·8%	97·1%	67	0	15	1	93·8%	100%
ATXN2	58	0	8	1	88·9%	100%	58	0	8	1	88·9%	100%
ATXN3	52	0	5	0	100%	100%	52	0	5	0	100%	100%
ATXN7	52	0	1	0	100%	100%	52	0	1	0	100%	100%
C9orf72	62	0	6	0	100%	100%	62	0	6	0	100%	100%
CACNA1A	56	0	10	0	100%	100%	56	0	10	0	100%	100%
DMPK	0	0	42	0	100%	100%	0	0	42	0	100%	100%
FMR1	22	1	20	0	100%	95·7%	23	0	20	0	100%	100%
FXN	46	0	13	3	81·3%	100%	46	0	16	0	100%	100%
HTT	65	2	45	1	97·8%	97%	67	0	46	0	100%	100%
TBP	57	0	2	0	100%	100%	57	0	2	0	100%	100%
**TOTAL**	**606**	**4**	**177**	**6**	**96·7%**	**99·3%**	**611**	**0**	**180**	**2**	**98·9%**	**100%**

Table S7. **Concordance between PCR and ExpansionHunter at repeat length, when considering repeats smaller or equal than the read-length (150 bp)**. count_concordant = total number of alleles whose length predicted by WGS matches the PCR with an error or +/- 1 repeat error; count_discordant = total number of alleles whose length predicted by whole genome sequencing does not match the PCR. Data based on ExpansionHunter v3.1.2. See Table S4 for full details.

	**WGS output**		
	count_concordant	count_discordant	concordance
AR	36	1	92·3%
ATN1	50	1	92·6%
ATXN1	101	9	91·8%
ATXN2	74	2	97·4%
ATXN3	51	1	92·8%
ATXN7	48	1	97·9%
C9orf72	125	5	95·4%
CACNA1A	63	1	98·4%
DMPK	13	7	61·9%
FMR1	31	8	80%
FXN	60	5	89·5%
HTT	145	8	95·5%
TBP	39	17	69·6%
**OVERALL**	836	66	**92·7%**

Table S8. **Repeat sizes predicted by whole genome sequencing for alleles classified as non-expanded, premutation or full mutation by PCR for each locus**. Repeat-size estimates by ExpansionHunter version 3.1.2 after visual inspection. For each locus and classification (non-expanded, premutation, full mutation) the mean, standard deviation, median, and quantiles of estimated repeat-sizes are calculated. For *DMPK*, small STRs were defined with size < 110, expansions >=110 and <130, and large STRs were defined with size >=130 repeats.

**Locus**	**PCR classification**	**WGS repeat sizes**	
		**Mean [SD] (range: MIN-MAX)**	**Median**
AR	non-expanded (<35)	22·5 [SD 3·7](16-28)	22
	premutation (35-37)	NA	NA
	full mutation (>37)	50 [SD 2·9] (46-54)	50
ATN1	non-expanded (<35)	17·7 [SD 4·1] (12-28)	19
	premutation (35-47)	NA	NA
	full mutation (>47)	60·7 [SD 2·9] (58-66)	58
ATXN1	non-expanded (<36)	30·5 [SD 1·5] (27-35)	30
	premutation (36-43)	36·3 [SD 0] (36-36)	36
	full mutation (>43)	43 [SD 14·1]	43
ATXN2	non-expanded (<32)	22 [SD 0] (17-31)	22
	premutation (32-32)	32 [SD 0]	32
	full mutation (>32)	38·3 [SD 2·6] (34-41)	40
ATXN3	non-expanded (<44)	18·8 [SD 3·3] (11-32)	20
	premutation (44-59)	NA	NA
	full mutation (>59)	59·7 [SD 2·6] (57-64)	58
ATXN7	non-expanded (<18)	10·5 [SD 0·7] (7-13)	10
	premutation (18-35)	NA	NA
	full mutation (>35)	84 [SD 67·7] ()	84
CACNA1A	non-expanded (<18)	11·7 [SD 1·5] (7-14)	12
	premutation (18-19)	18 [SD 0]	18
	full mutation (>19)	22 [SD 0]	22

C9orf72	non-expanded (<31)	4·5 [SD 3·6] (2-24)	2
	premutation (31-59)	36 [SD 8·9]	36
	full mutation (>59)	63·6 [SD 28·6] (42-117)	53
DMPK	non-expanded (<50)	10·8 [SD 6·1] (5-21)	12
	small EXP	102·5 [SD 51] (52-150)	109
	EXP	125 [SD 17·8] (71-173)	131·5
FMR1	non-expanded (<45)	30 [SD 1·9] (20-57)	32·6
	premutation (55-200)	84 [SD 8·5] (72-95)	85
	full mutation (>200)	92·6 [SD 17·8](70-131)	89
FXN	non-expanded (<45)	11·3 [SD 0·7] (3-32)	9
	premutation (45-65)	NA	NA
	full mutation (>65)	115·3 [SD 25·9] (52-197)	111
HTT	non-expanded (<36)	21·4 [SD 6·7] (12-35)	19
	premutation (36-39)	36 [SD 0] (36-36)	36
	full mutation (>39)	49·6 [SD 3·7] (40-97)	45
TBP	non-expanded (<42)	36·8 [SD 1·3] (27-41)	37
	premutation (42-48)	43 [SD 0·7] (42-44)	43
	full mutation (>48)	NA	NA

Table S9. Total number of repeat expansions called before and after visual inspection, repeats tested by PCR and repeats confirmed by locus in each virtual panel (A-D). Breakdown of repeat expansion called before (`RE called`) and after visual inspection (`RE after inspection`), `RE orthogonally tested` (repeat expansion with DNA available that has been tested by PCR) and orthogonally confirmed (`Confirmed RE`) in the four panels and by locus. The columns `RE called` and `RE after inspection` include the total number of genomes with a repeat expansion estimated by ExpansionHunter before and after visual inspection respectively, `RE orthogonally tested` contains the total number of patients tested by PCR, and `Confirmed RE` includes the total number of genomes after validating by PCR.

Due to formatting and display issues, as well as facilitating a more detailed examination, this table is available at the following web address: https://drive.google.com/file/d/1AIBpg71J3OoG6WdOrlaVO6-fDkP3uYYD/view?usp=sharing

Table S10. 100,000 Genomes Project patients with pathogenic expansions identified in this study and confirmed by PCR. For each repeat expansion confirmed, information regarding the clinical presentation together with biological sex, age range, the gene where the repeat expansion was confirmed, and repeat-sizes for both alleles (`A1` and `A2`) are shown. The numbers in alleles correspond to the number of repeats estimated by ExpansionHunter v2.5.5. HPO terms are included for each patient. `Contribution to the phenotype` states the contribution of each repeat expansion to the patient's phenotype following review by the local clinician; `Report issued` specifies whether a diagnostic report has been returned.

Due to formatting and display issues, as well as facilitating a more detailed examination, this table is available at the following web address:
https://drive.google.com/file/d/13kpkou_VESgZigt7zcMhWdhsvlFxLRSR/view?usp=sharing

Table S11. **Total number of 24 genomes not validated by PCR because of lack of DNA**. **Figure S7** contains the pileup graphs corresponding to each case. The repeat-size estimated by Expansion Hunter version 2.5.5 before and after visual inspection are provided, showing good pileups and potentially positive cases.

Due to formatting and display issues, as well as facilitating a more detailed examination, this table is available at the following web address:
https://drive.google.com/file/d/19TXCnhFPybWzsLsnmOgVBPQre38G6xom/view?usp=sharing

Table S12. Supplementary clinical details for Panel B, based on HPO terms.

Clinical presentation	Patients tested	Repeat expansion tested	Repeat expansion called	Repeat expansion after visual inspection	Repeat expansion tested	Confirmed repeat expansion
**COMPLEX NEURODEVELOPMENTAL: paediatric patients with intellectual disability (ID)**	**2743**	*ATN1, ATXN1, ATXN2, ATXN3, ATXN7, CACNA1A, HTT*	14	9	8	8
ID AND seizures only	1048		2	2	2	2
ID AND dystonia only	22		1	0	0	0
ID AND ataxia only	116		0	0	0	0
ID AND spastic paraplegia only	76		0	0	0	0
ID AND optic neuropathy OR retinopathy only	10		0	0	0	0
ID AND white matter abnormalities only	91		0	0	0	0
ID AND muscular weakness or hypotonia only	117		0	0	0	0
ID and at least one of the above	2743		14	9	8	8
ID and at least two of the above	1022		11	7	6	6
ID and at least three of the above	615		8	6	4	4
ID and at least four or more of the above	317		1	1	1	1

Table S13. Curated coordinates for json files (ExpansionHunterv2.5.5). Genomic coordinates defined for the region where the repeat motif is located in each gene. Different specifications have been used for GRCh37 and GRCh38 human genome assemblies when running ExpansionHunter v2.5.5.

**STR gene**	**Repeat motif**	**Coordinates GRCh37**	**Coordinates GRCh38**
AR	CAG	X:66765160-66765225	chrX:67545318-67545383
ATN1	CAG	12:7045892-7045936	chr12:6936729-6936773
ATXN1	CTG	6:16327867-16327953	chr6:16327636-16327722
ATXN2	CTG	12:112036755-112036823	chr12:111598951-111599019
ATXN3	CTG	14:92537355-92537396	chr14:92071011-92071052
ATXN7	CAG	3:63898362-63898391	chr3:63912686-63912715
C9orf72	GGCCCC	9:27573527-27573544<br>+off target regions	chr9:27573529-27573546<br>+ off target regions
CACNA1A	CTG	19:13318673-13318711	chr19:13207859-13207897
DMPK	CAG	19:46273463-46273522	chr19:45770205-45770264
FMR1	CGG	X:146993569-146993628<br>+ off target regions	chrX:147912051-147912110<br>+off target regions
FXN	GAA	9:71652203-71652220	chr9:69037287-69037304
HTT	CAG	4:3076604-3076660	chr4:3074877-3074933
TBP	CAG	6:170870996-170871109	chr6:170561908-170562021

Table S14. Curated coordinates for json files (ExpansionHunterv3.1.2). Genomic coordinates defined for the region where the repeat motif is located in each gene. Different specifications have been used for GRCh37 and GRCh38 human genome assemblies when running ExpansionHunter v3.1.2. Coordinates corresponding to *AR, ATN1,* and *ATXN3* have been updated from the GitHub repository (https://github.com/Illumina/ExpansionHunter) for this analysis to match the repeats targeted by PCR.

STR gene	Repeat motif	Coordinates GRCh37	Coordinates GRCh38
AR	(GCA)*	X:66765161-66765227	chrX:67545319-67545385
ATN1	(CAG)*	12:7045891-7045936	chr12:6936728-6936773
ATXN1	(TGC)*	6:16327867-16327954	chr6:16327636-16327723
ATXN2	(GCT)*	12:112036753-112036822	chr12:111598949-111599018
ATXN3	(GCT)*	14:92537344-92537386	chr14:92071000-92071042
ATXN7	(GCA)*(GCC)+	3:63898360-63898390<br>3:63898390-63898402	chr3:63912684-63912714<br>chr3:63912714-63912726
C9orf72	(GGCCCC)*	9:27573526-27573544 + off target regions	chr9:27573528-27573546 + off target regions
CACNA1A	(CTG)*	19:13318672-13318711	chr19:13207858-13207897
DMPK	(CAG)*	19:46273462-46273522	chr19:45770204-45770264
FMR1	(CGG)*	chr9:27573528-27573546 + off target regions	chrX:147912050-147912110 + off target regions
FXN	(A)*(GAA)*	9:71652177-71652202<br>9:71652202-71652220	chr9:69037261-69037286<br>chr9:69037286-69037304
HTT	(CAG)*CAACAG(CCG)*	4:3076603-3076660<br>4:3076666-3076693	chr4:3074876-3074933<br>chr4:3074939-3074966
PPP2R2B	(GCT)*	5:146258290-146258320	chr5:146878727-146878757
TBP	(GCA)*	6:170870991-170871105	chr6:170561906-170562017

Table S15. Table 2 by patient ethnicity. The self-reported ancestry (called internally `participant_ethnic_category`) has been used. Each group has been re-coded as follows: Asian as `Asian or Asian British: Pakistani`, `Asian or Asian British: Indian`, `Asian or Asian British: Any other Asian background`, `Asian or Asian British: Bangladeshi`. African as `Black or Black British: African`, `Black or Black British: Caribbean`, `Black or Black British: Any other Black background`. Multi-ethnic as `Mixed: Any other mixed background`, `Mixed: White and Asian`, `Mixed: White and Black Caribbean`, `Mixed: White and Black African`; Other as `Other Ethnic Groups: Chinese`, `Other Ethnic Groups: Any other ethnic group`; European as `White: Irish`, `White: British`, `White White: British`, `White: Any other White background`.

	**Asian**	**African**	**Multi-ethnic**	**Not stated**	**Other**	**European**
**Overall**	0.1	0	0	0.2	0	0.7
Hereditary ataxia	0.1	0	0	0.1	0	0.8
Hereditary spastic paraplegia	0.2	0	0	0.1	0	0.7
Early onset and familial Parkinson's Disease	0.1	0	0	0.1	0	0.8
Complex Parkinsonism (includes pallido-pyramidal syndromes)	0.1	0.05	0.02	0.1	0.03	0.7
Early onset dystonia	0.1	0	0.03	0.05	0.02	0.7
Early onset dementia	0.1	0.05	0.025	0.1	0.025	0.7
Amyotrophic lateral sclerosis or motor neuron disease	0.2	0.1	0	0.2	0	0.5
Charcot-Marie-Tooth disease	0.1	0	0	0.1	0	0.8
Ultra-rare undescribed monogenic disorders	0.1	0	0	0.2	0	0.7
Congenital myopathy	0.1	0.05	0.025	0.1	0.025	0.7
Distal myopathies	0.1	0.025	0.025	0.1	0.05	0.7
Congenital muscular dystrophy	0.1	0.03	0	0.1	0.07	0.7
Skeletal muscle channelopathy	0.1	0	0	0.1	0	0.8
Intellectual disability	0.1	0	0	0.2	0	0.7

TableS16. Diagnostic accuracy of the pipeline by ExpansionHunterv2.5.5. TN= true negative; FP = false positive; TP = true positive; FN = false negative

	EHv2.5.5					
	**TN**	**FP**	**TP**	**FN**	**Sensitivity**	**Specificity**
AR	36	0	7	0	100%	100%
ATN1	91	0	3	0	100%	100%
ATXN1	145	5	15	1	93·8%	96·7%
ATXN2	123	2	8	1	88·9%	98·4%
ATXN3	107	2	5	0	100%	98·2%
ATXN7	105	0	1	0	100%	100%
C9orf72	130	0	6	0	100%	100%
CACNA1A	122	0	10	0	100%	100%
DMPK	42	0	42	0	100%	100%
FMR1	45	0	20	0	100%	100%
FXN	69	1	51	3	94·4%	98·6%
HTT	179	1	46	0	100%	99·4%
TBP	114	2	2	0	100%	98·3%
**TOTAL**	**1308**	**13**	**216**	**5**	**97·7%**	**99%**
